# Ligand Structure Determines Nanoparticles' Atomic Structure, Metal-Ligand Interface and Properties

**DOI:** 10.3389/fchem.2018.00330

**Published:** 2018-08-07

**Authors:** Milan Rambukwella, Naga Arjun Sakthivel, Jared H. Delcamp, Luca Sementa, Alessandro Fortunelli, Amala Dass

**Affiliations:** ^1^Department of Chemistry and Biochemistry, University of Mississippi, Oxford, MS, United States; ^2^CNR-ICCOM and IPCF, Consiglio Nazionale delle Ricerche, Pisa, Italy

**Keywords:** ligand effect, nanoparticle atomic structure, metal ligand interface, ligand-ligand interaction, nanoparticle synthesis

## Abstract

The nature of the ligands dictates the composition, molecular formulae, atomic structure and the physical properties of thiolate protected gold nanomolecules, Au_n_(SR)_m_. In this review, we describe the ligand effect for three classes of thiols namely, aliphatic, AL or aliphatic-like, aromatic, AR, or bulky, BU thiol ligands. The ligand effect is demonstrated using three experimental setups namely: (1) The nanomolecule series obtained by direct synthesis using AL, AR, and BU ligands; (2) Molecular conversion and interconversion between Au_38_(S-AL)_24_, Au_36_(S-AR)_24_, and Au_30_(S-BU)_18_ nanomolecules; and (3) Synthesis of Au_38_, Au_36_, and Au_30_ nanomolecules from one precursor Au_n_(S-glutathione)_m_ upon reacting with AL, AR, and BU ligands. These nanomolecules possess unique geometric core structure, metal-ligand staple interface, optical and electrochemical properties. The results unequivocally demonstrate that the ligand structure determines the nanomolecules' atomic structure, metal-ligand interface and properties. The direct synthesis approach reveals that AL, AR, and BU ligands form nanomolecules with unique atomic structure and composition. Similarly, the nature of the ligand plays a pivotal role and has a significant impact on the passivated systems such as metal nanoparticles, quantum dots, magnetic nanoparticles and self-assembled monolayers (SAMs). Computational analysis demonstrates and predicts the thermodynamic stability of gold nanomolecules and the importance of ligand-ligand interactions that clearly stands out as a determining factor, especially for species with AL ligands such as Au_38_(S-AL)_24_.

## Introduction

The effect of the nature of a ligand on passivated nanoparticles (NPs) is not well understood. A variety of nanoparticles that are monodispersed in size (Alvarez et al., 1997; Love et al., 2005; Li and Jin, 2013; Knoppe and Bürgi, 2014; Weissker et al., 2014) are available, through the advancement of synthetic protocols. This is important since lack of atomic monodispersity can, limit the use of NPs in their applications. Recently, a wide variety of atomically precise gold nanomolecules (AuNMs) (Whetten et al., 1996; Daniel and Astruc, 2004; Tsukuda and Häkkinen, 2015) and NPs, with unique-structures and properties have been synthesized using robust synthetic protocols (Qian et al., 2009b; Wu et al., 2009; Udayabhaskararao and Pradeep, 2013; Zhang et al., 2016; Rambukwella and Dass, 2017; Theivendran and Dass, 2017). These are used in a wide range of applications, such as catalysis (Qian et al., 2012; Li and Jin, 2013; Kwak et al., 2017), biosensing (Saha et al., 2012; Kwak et al., 2014), supra molecular chemistry (Daniel and Astruc, 2004; Abbas et al., 2016), and therapeutic agents (Thakor et al., 2011). In contrast to AuNPs (diameter 3–100 nm) with surface plasmon resonance and high degree of polydispersity in size (**Scheme 2a**), AuNMs (diameter < 2 nm) have characteristics attributed to their atomic monodispersity (±0 atom) and size-dependent molecule-like properties (Murray, 2008). Among the highly investigated AuNMs, crystallographically characterized, examples include Au_25_(SR)_18_, Au_30_(SR)_18_, Au_36_(SR)_24_, Au_38_(SR)_24_, Au_102_(SR)_44_, Au_130_(SR)_50_, Au_133_(SR)_52_ and Au_279_(SR)_84_ (Jadzinsky et al., 2007; Heaven et al., 2008; Zhu et al., 2008; Qian et al., 2010; Zeng et al., 2012; Crasto et al., 2014b; Nimmala et al., 2014b; Chen et al., 2015b; Dass et al., 2015; Sakthivel et al., 2017) where, SR represents physicochemically different thiolate ligands.

Typically, thiolate ligands are implemented as a self-assembling monolayer (SAM) (Azcárate et al., 2013; Indrasekara et al., 2014; Burrows et al., 2016), that governs the atomic structure, stability, electrochemical properties and functionality of the as-synthesized nanoparticles. Thiolate protected AuNMs and AuNPs are comprised of three main structural components: an inner metallic-core, metal-thiolate interfaces composed of staple motifs and outermost thiolate surfaces that governs characteristics such as solubility. The surface of these AuNMs is surrounded by a variety of staple motifs (Jiang et al., 2008). For instance, a combination of directly linked gold-thiolate motifs (Jiang et al., 2008; Indrasekara et al., 2014), monomeric staples (RS-Au-SR), dimeric staples (RS-Au-SR-Au-SR) and trimeric staples (RS-Au-SR-Au-SR-Au-SR) have been identified for AuNMs with physicochemically different ligands (Figure S1) (Jadzinsky et al., 2007; Heaven et al., 2008; Qian et al., 2010; Nimmala et al., 2014b; Dass et al., 2016). The bridge between the surface structure assembly and how it relates to surface chemistry of the metal-thiolate of nanocomposites and their structural stability and selectivity remains unclear to date (Zeng et al., 2012).

Density functional theory (DFT) based investigation on photoluminescent Au_18_(SR)_14_ clusters, was reported by Tlahuice-Flores, where, they study the effect of 6 physicochemically different ligands (Tlahuice-Flores, 2016). They found that presence of different functional groups, such as phenyl rings, nitro groups or alkyl group, plays a key role on the structure and properties of Au_18_(SR)_14_. Major structural distortions in Au_18_(SR)_14_ clusters were observed with para-mercaptobenzoic acid and para-nitrobenzenethiol whereas, with -SCH_3_, 4-tert-butylbenzenethiol (TBBT, HSPh-*t*Bu), thiophenol and cyclohexanethiol ligands, similar structure and ligand orientation were observed. Another study by Tlahuice-Flores et al. reported ligand induced structural distortions in Au_25_(SR)_18_ clusters (Tlahuice-Flores et al., 2013), where they investigated a set of 11 ligands. From these ligands, they found that para-substituted thiophenolate ligands with electron-withdrawing groups induced major structural distortions in the Au_25_S_18_ framework resulting in less symmetric structures. Interestingly, the thiolate ligands with low polarity such as –SH, –SCH_3_, and –SC_6_H_13_ as well as the –S(CH_2_)_2_Ph (phenylethane thiol) retain the C_*i*_ symmetry of the total crystal structure. As a result, a decrease in the HOMO-LUMO gap was found to be more evident in the case of electron withdrawing ligand protected Au_25_(SR)_18_. Thus, ligand effects are not necessarily due to only the ligands' bulkiness but also due to the aromaticity and electronic nature of the ligand structure. Experimentally, we have shown that in the presence of the aromatic TBBT ligand Au_144_(SCH_2_CH_2_Ph)_60_ transforms to a new core-size to give Au_133_(SPh-*t*Bu)_52_ (Nimmala et al., 2015). We hypothesize the effect of aromatic phenyl rings contribute a favorable inter-ligand interaction while para-tertiary groups create steric repulsion and kinetic effects and trigger the core-size conversion to Au_133_(SPh-*t*Bu)_52_. In the presence of only aromatic ligands (without drastic sterically crowding substitutes, –tBu) such as thiophenol, it was observed that the Au_144_(SCH_2_CH_2_Ph)_60_ NMs transforms to Au_99_(SPh)_42_ (Nimmala and Dass, 2014) which suggest that the bulk *tert*-butyl group of TBBT plays a key role in determining the atomic structure by changing ligand-ligand interactions. Recent reports on Au_38_(SCH_2_CH_2_Ph)_24_ NMs revealed that ligands can induce core-size conversions on relatively small NMs as well. In the presence of TBBT ligands Au_38_(SCH_2_CH_2_Ph)_24_ is core size converted to give Au_36_(SPh-*t*Bu)_24_ NMs (Zeng et al., 2013) and in the presence of bulky tert-butylthiol ligands Au_30_(S-tBu)_18_ NMs (Rambukwella et al., 2017b). Furthermore, recently we have demonstrated ligand induced molecular interconversion (Dass et al., 2017) between Au_36_(SPh-X)_24_ (where X = –H or –tBu,) and Au_30_(S-tBu)_18_ NMs. These reports demonstrated that by controlling the ligand-ligand interaction by means of steric bulk and aromaticity of the thiolate ligand, it is possible to interconvert between similar size physicochemically different AuNMs. Therefore, physicochemically different thiolate ligands have been widely used to control the atomic structures and ligand environments of AuNMs and tune unique properties in nanoparticles.

Maran et al. have reported the effect of alkyl ligand length on electron transfer reactions in Au_25_(SC_n_H_2n+1_)_18_ NMs with n = 2, 4, 6, 8, 10, 12, 14, 16, 18 (Antonello et al., 2014). The results show a difference in electron transfer rates between short ligands and long ligands. Cirri et al. have also showed that chain length of the ligands has a direct control over electronic properties of AuNMs and the degree of charge transfer can be controlled by the difference between the dielectric constant of the solvent and the surface ligand of the AuNMs (Cirri et al., 2016). Another study carried out on [Au_25_(SCH_2_CH_2_Ph)_18_]^0^ NMs by Agrachev et al. showed that magnetism can be controlled from paramagnetic to superparamagnetic to ferromagnetic as a function of the aggregation state of the clusters (Agrachev et al., 2017). Recently, we have demonstrated that in contrast to AL ligands, AR ligands induces a bathochromic shift in the Au_38_(SR)_24_ absorption spectra and reduces the electrochemical band gap (Rambukwella et al., 2017a).

Interestingly, Wang et al. demonstrated the importance of halides in the formation of large bimetallic Au_80_Ag_30_ NMs (Zeng et al., 2016). In their study, a total of 9 chloride atoms were found coordinated to the third shell and each chloride atom was found bridged to two Ag atoms. In contrast to larger NMs, Zhu et al. reported a comparison of aliphatic ligand against aromatic ligand protected small Au_15_Ag_3_ NMs, where different structural, electronic and optical properties were observed with structurally different thiolate ligands (Kang et al., 2017). Recently, it has been shown that TBBT thiol protected Au_28_(SPh-*t*Bu)_20_ can be interconverted to cyclohexyl thiol protected Au_28_(SR)_20_ (Chen et al., 2016b) and Au_24_L_20_ (L-ligand) with phenylethane thiol and selenophenol ligand (Song et al., 2014) can be synthesized, but the atomic structure and properties of those NMs were found to be different. Han et al. theoretically investigated aromatic and aliphatic thiol ligand effects on Au_25_, Au_38_, and Au_102_ NMs and they reported aliphatic thiols stabilize the NMs more than the aromatic ligands both thermodynamically and electrochemically (Jung et al., 2012). The authors also reported that the stabilization energy of NMs varies depending on ligand structure regardless of inter-ligand interaction, system size and shape. Similarly, it was reported that subtle changes in the structure of the surface ligand would trigger formation of NMs (Chen et al., 2015a,b) with completely different atomic structures and properties. However, the underlying fundamental aspects of the ligand structure dependence on NMs' atomic structure remains to be not well established.

A major advancement in NM research took place with the pioneering work by Kornberg et al. in the discovery of the crystal structure of para-mercaptobenzoic acid (p-MBA) protected Au_102_(p-MBA)_44_ NMs. The stability of the Au_102_ system has been attributed to the “superatom chemistry” of the nanomolecule (Jadzinsky et al., 2007). The reasoning behind the superatom electronic configuration is analogous to the inert electronic shell closing observed and attained by gas-phase atoms and molecules. It is assumed that each gold atom contributes one valence electron to the molecular orbitals and each thiolate ligand localizes one electron thus, Au_102_(p-MBA)_44_ NMs possess 58 electrons (102-44 = 58) with superatom electronic configuration (2, 8, 20, 40, 58, 84). While well studied AuNMs systems such as Au_10_(SR)_10_, Au_15_(SR)_13_, Au_25_${\left(\text{SR}\right)}_{18}^{-}$, Au_144_(SR)_60_ obeys superatom theory, stability of other AuNMs such as Au_30_, Au_36_, Au_38_ and Au_133_ does not fit in the superatom magic electron shell closing trend. Superior stability associated with AuNMs that deviate from the electronic structural integrity governed by superatom theory, which suggests the right perspective of AuNMs structural selectivity and stability is being governed by the ligand structure and geometry.

**Scheme 1:**
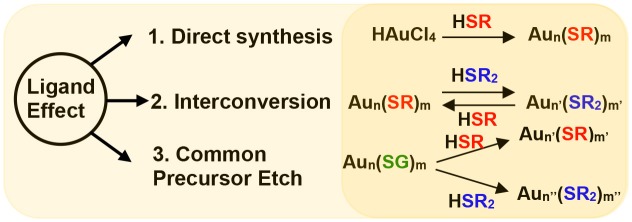
Ligand effect demonstrated by three experimental setups namely; via 1. direct synthesis of NMs, 2. interconversion of NMs and, 3. etching of a common precursor (Au_n_(SG)_m_) with different thiols (HSR and HSR_2_).

In this review we have investigated the bulkiness and electronic nature of surface ligands on the formation of AuNMs and their influence on physicochemical properties using three experimental setups approaches namely: (1) The nanomolecule series obtained by direct synthesis of AuNMs employing AL, AR, BU ligands; (2) Molecular conversion and interconversion between Au_38_(S-AL)_24_, Au_36_(S-AR)_24_, and Au_30_(S-BU)_18_ nanomolecules; and (3) Synthesis of Au_38_, Au_36_, and Au_30_ nanomolecules from a common precursor Au_n_(S-glutathione)_m_ upon reacting with AL, AR, and BU ligands (Scheme 1). These experimental approaches unveil fundamental aspects of surface ligand structure and atomic structure of AuNMs and their physicochemical properties. This review emphasizes the consideration of ligand effects in the design and synthesis of novel NMs.

## Ligand effect demonstrated by direct synthesis

**Scheme 2:**
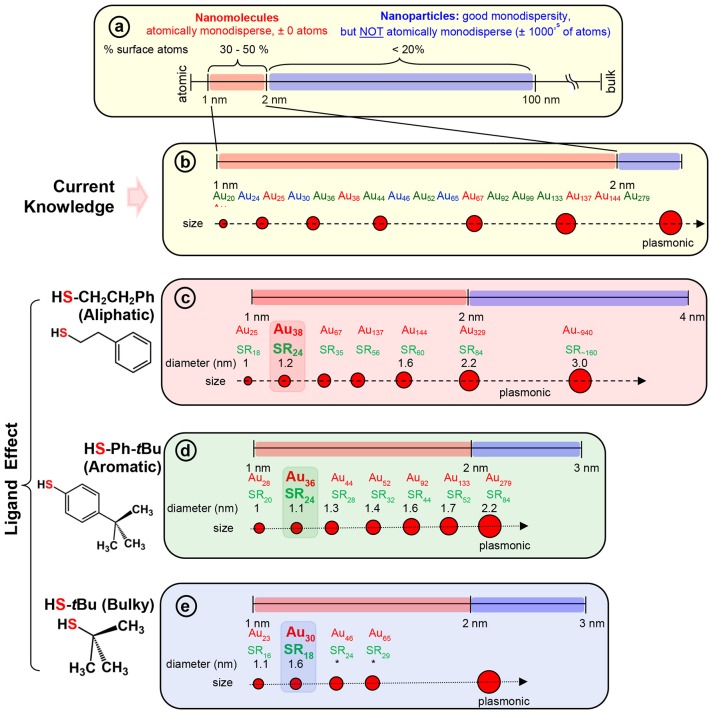
**(a)** The scheme shows the 1–100 nm size regime with atomically precise NMs in the 1–2 nm region, and NPs in the 2–100 nm regime (where, very good monodispersity in size has been achieved, but atomic composition can vary by ± 1000's of atoms). Examples of widely investigated NMs with three classes of thiolate ligand are given under each series. **(b)** Widely investigated thiolate protected gold NM systems belonging to each series. NMs with; **(c)** aliphatic and aliphatic-like ligand, HSCH_2_CH_2_Ph, **(d)** aromatic ligand, HSPh-*t*Bu, and **(e)** bulky ligand, HS-*t*Bu are illustrated under each scheme. The thermodynamically most stable NM in the aliphatic, aromatic, and bulky series namely, Au_38_(SCH_2_CH_2_Ph)_24_, Au_36_(SPh-*t*Bu)_24_, and Au_30_(S-*t*Bu)_18_ NMs respectively, are highlighted. Asterix indicates unknown diameter of the NM due to lack of crystal structure.

The two-phase Brust method (Brust et al., 1994) and methods derived from Brust synthesis have been widely implemented to synthesize a wide range of AuNMs (Scheme 2b). The strong Au-S covalent bond (Kokkin et al., 2015) between thiolate ligands and surface gold atoms makes synthetic protocols highly robust for thiolate protected AuNMs. The surface orientations of each thiol vary and for instance when the steric requirements of the ligand preclude the ordering found for the aliphatic thiolate structures, other ordering are found in SAMs (Love et al., 2005). In contrast to aliphatic thiols, aromatic thiols such as *p*-biphenylthiols, *p*-terphenylthiols, and oligo (phenylene ethynylene) thiols are found to have a slightly less bent orientation on a Au(111) surface. Most importantly, a wide variety of AuNMs has been reported mainly by altering the structure of thiolate ligands. Various size-dependent properties are found in these AuNMs protected by different thiolate ligands. Interestingly, based on the physicochemical nature of the thiol, we have observed that certain class of thiolate ligands exclusively forms a unique series of AuNMs. Based on these experimental observations, thiolate ligands can be categorized into three main classes, namely; aliphatic, aromatic and bulky (Scheme 2c,d and Figure S1) thiolate ligands, where they differ from each other at the sulfur-carbon bond. For instance, aliphatic ligands possess a primary carbon atom immediately bonded to the sulfur atoms, aromatic ligands have carbon atom in aromatic ring bonded to the sulfur atom and bulky thiols have a tertiary carbon atom immediately bonded to the sulfur atom.

Among the AL ligands, phenylethane thiol (HSCH_2_CH_2_Ph, PET) is the most widely used and studied ligand by Murray and co-workers, followed by others. To date, a series of highly robust and thermodynamically stable NMs (Scheme 2c) such as Au_25_(SCH_2_CH_2_Ph)_18_, Au_38_(SCH_2_CH_2_Ph)_24_, and Au_144_(SCH_2_CH_2_Ph)_60_ have been reported with the PET ligand (Zhu et al., 2008; Qian and Jin, 2009; Qian et al., 2009b, 2010) and other physicochemically similar ligands such as ethanethiol (Dainese et al., 2014), hexanethiol (García-Raya et al., 2009; Stellwagen et al., 2012), octanethiol (Stellwagen et al., 2012), and dodecanethiol (Toikkanen et al., 2008; Qian et al., 2009a; Stellwagen et al., 2012). Interestingly, we have observed that NMs such as Au_21_(SR)_15_, Au_30_(SR)_18_, Au_36_(SR)_24_, Au_133_(SR)_52_ etc. are not formed or have not been reported with AL ligands. Thus, AL and aliphatic-like ligands govern the exclusive formation of a unique series of NMs (Scheme 2c and Table 1).

**Table 1:** Three physicochemically different series of NMs observed with class of aliphatic, aromatic and bulky thiolate ligands.

	**Size**	**Citations**
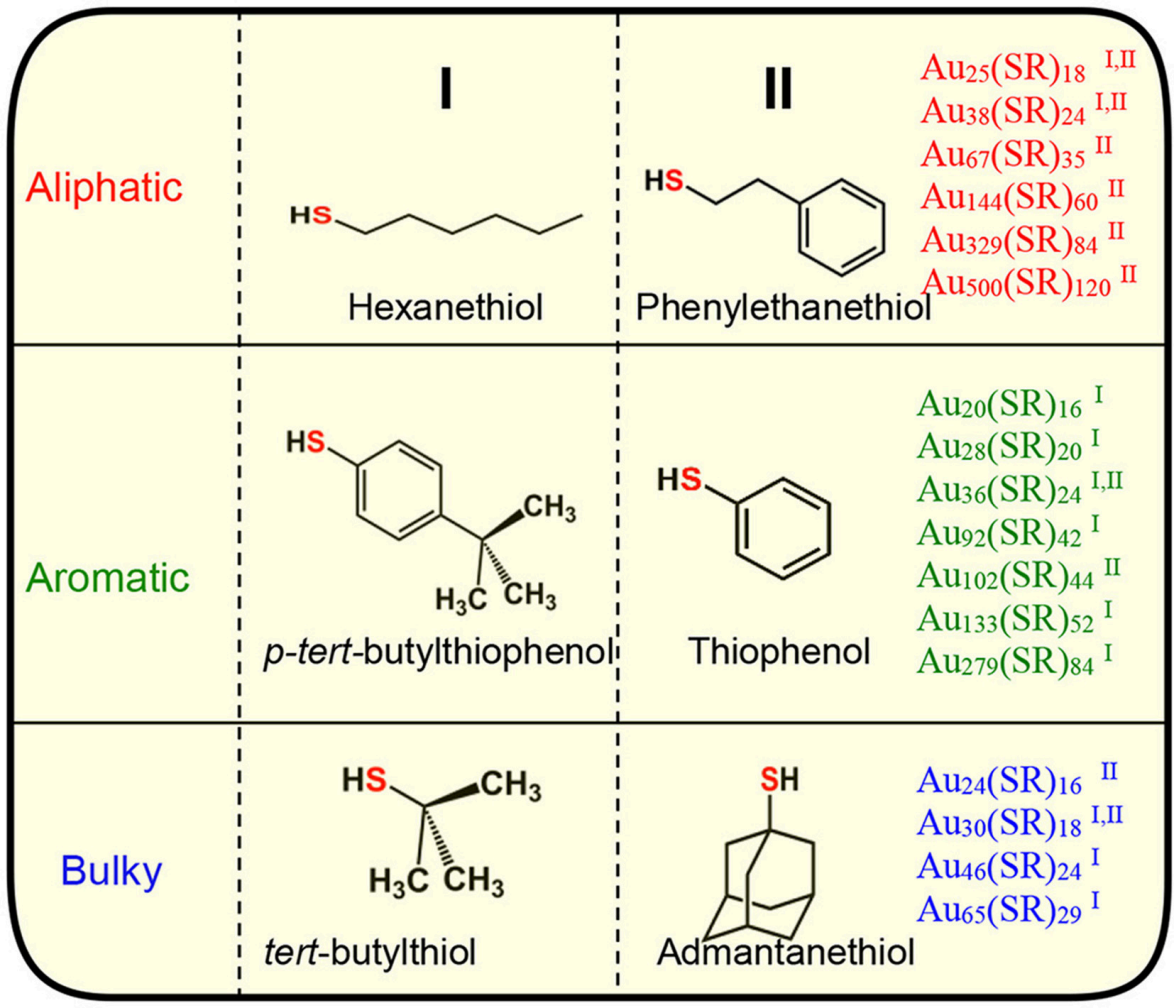	**SCH_2_CH_2_Ph (Aliphatic)**	
	Au_25_(SR)_18_	*J. Am. Chem. Soc*., 2007, 16209
	Au_38_(SR)_24_	*J. Am. Chem. Soc*., 2004, 6193
	Au_67_(SR)_35_	*J. Phys. Chem. A*, 2013, 504
	Au_137_(SR)_56_	*Chem. Commun.*, 2014, 9895
	Au_144_(SR)_60_	*J. Phys. Chem. C*, 2009, 5035
	Au_329_(SR)_84_	*Anal. Chem*., 2014, 4227
	Au_~940_(SR)_~160_	*ACS Nano*, 2014, 6431
	**-S-Ph-*t*Bu (Aromatic)**	
	Au_28_(SR)_20_	*J. Am. Chem. Soc*., 2013, 10011
	Au_36_(SR)_24_	*J. Am. Chem. Soc*., 2011, 9175
	Au_44_(SR)_28_	*J. Am. Chem. Soc*., 2005, 13750
	Au_52_(SR)_32_	*Nanoscale*, 2016, 1299
	Au_92_(SR)_44_	*J. Am. Chem. Soc*., 2016, 8710
	Au_102_${\left(\text{SR}\right)}_{18}^{-}$	*Chemtracts*, 2007, 308
	Au_133_(SR)_52_	*J. Am. Chem. Soc*., 2015, 4610
	Au_279_(SR)_84_	*J. Am. Chem. Soc*., 2017, 15450
	**-S-*t*Bu (Bulky)**	
	Au_24_(SR)_16_	*J. Am. Chem. Soc*., 2014, 14933
	Au_30_(SR)_18_	*J. Am. Chem. Soc*., 2014, 5000
	[198]Au_46_(SR)_24_	*J. Phys. Chem. C*, 2018
	Au_65_(SR)_29_	*J. Phys. Chem. C*, 2018

**Figure 1:**
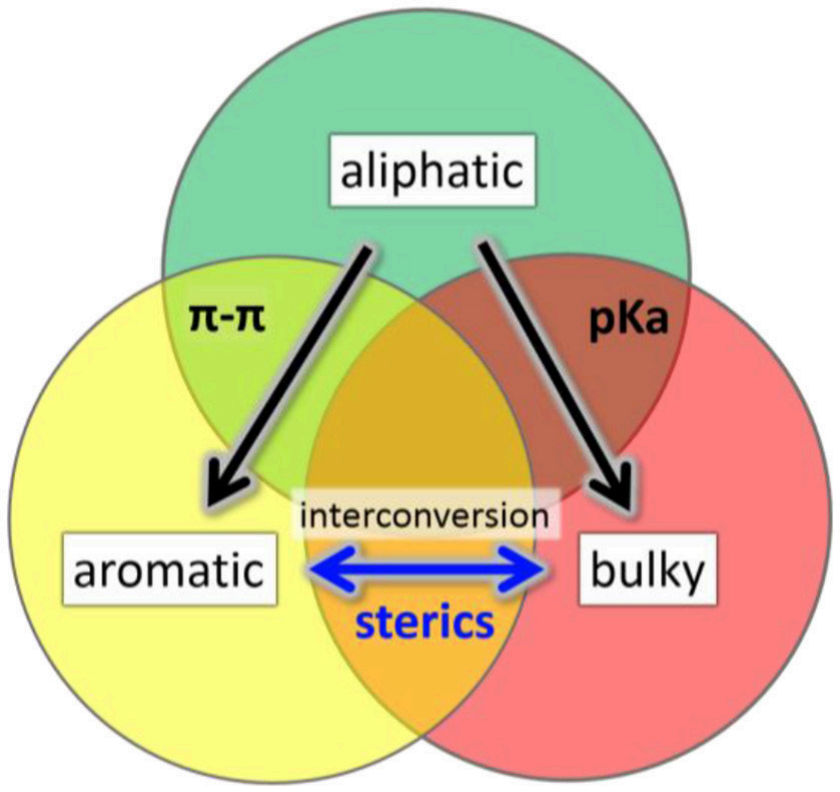
Illustration of overlapping characteristics (π-π interactions, pka values, steric bulk) of the primary, aromatic, and BU tertiary ligands in this work. Arrows show the direction of conversion for Au NMs using these ligand classes.

We have investigated the effect of physicochemically different ligands on AuNMs by comparing an aliphatic-like thiol ligand (-S(CH_2_)_2_Ph), a BU thiol ligand (*tert*-butyl thiol), and an AR thiol (TBBT). Multiple factors have been examined including: (1) sterics based on ligand size, (2) ligand electron donor strength as evaluated by pKa values, and (3) π-π ligand interactions. These ligands were selected for comparison in AuNM core-size control since each of these ligands have at least one property examined which is similar to one of the other ligands and vastly different to the other (Figure 1). The potentially similar interactions are highlighted for each ligand in the overlapping circle areas in Figure 1. Specifically, the AL and AL-like thiol, -S(CH_2_)_2_Ph, has a very similar pKa to BU tert-butyl thiol (both at ~17 in DMSO) (Bordwell, 1988). However, the pKa of the aromatic ligand, TBBT, is ~10 in DMSO. This represents a 10^7^ more stabilized anion for the TBBT ligand upon deprotonation. Given the tremendously larger ability of the aromatic ligand to stabilize the sulfur lone electron pairs through resonance, a vastly diminished (by 7 orders of magnitude) sulfur electron donation strength is expected when compared with either of the alkyl ligands in organic solvent. This allows for the influence of the sulfur electron density to be probed with respect to the AuNMs structure. Thermodynamically, the AL and BU tertiary alkyl thiols would be predicted from first principles to form stronger Au-S bonds than aromatic thiols since aromatic thiols have competing π^*^ orbitals to accept electron density from the sulfur atom while alkyl thiols do not. The relatively larger amount of electron density at the sulfur atom of the alkyl thiols should promote stronger bonding to Au atoms. In support of this prediction, normalized cluster fragmentation energies of the AL, AR, and BU AuNMs are calculated as discussed below with reference to equations t1, t2, and t3, respectively. Also, average bond lengths for alkyl thiol Au-S bonds measured by x-ray crystallography are shorter than that of aromatic thiols by 0.008 Å (see discussion below). It is noteworthy that this bond length value does have some uncertainty associated with it based on the resolution for the three structures. This suggests a potentially thermodynamically more stable Au-S bond from the alkyl thiols. Providing aromatic thiols have weaker bonds, AuNMs based on AR thiols should be most easily converted to other AuNM core sizes. Importantly, the two ligands with similar pKas are vastly different in size and the TBBT ligand cannot have π-interactions, which allows for the probing of electron donor strength on the AuNM core-size primarily.

Concerning sterics, while A-values must be used with caution in assigning steric bulk, they do provide a general guideline for assigning steric influence of the ligand at the sulfur atom for our ligands. It should be noted that A-values apply best to cyclohexane systems, where diaxial conformation interactions are being quantified. A-values show the largest influence near the point where the group being analyzed is bound. When comparing the A-values of the groups (–(CH_2_)_2_Ph, TBBT and *tert*-butyl thiol) attached to the sulfur atom, it is important to remember that the atoms near the S atom will have the largest influence sterically according to the A-value scale. As an example, A-values decrease dramatically far from the attachment point (e.g., –Me is 1.70 and –Et is 1.75 despite a doubling of carbon atoms). With this in mind, the AL thiol would be predicted to have an A-value of 1.75 as the smallest ligand. Both the BU thiol (A-value of 4.5) and the AR thiol (A-value of 3.0) are considerably larger in terms of sterics. Given that the sulfur atom itself has an A-value of ~1.0, the primary group adds relatively little additional sterics. The other AR and BU tertiary alkyl thiol ligands provide significantly increased steric bulk beyond the size of the sulfur. Notably, the two largest ligands have vastly different pKas (AR vs. BU) and both cannot have π-π interactions (BU doesn't have an aryl group). Thus, this comparison allows for probing the steric influence with minimal input from other parameters.

Finally, concerning potential π-π interactions, two of the ligands utilize aryl groups, while BU *tert-*butyl thiol does not have an aryl group which precludes the influence of π-π ligand interactions. Importantly, the ligands which can have π-π interactions are vastly different in size and pKa values, which allows for the probing of π-interactions primarily on AuNM core-size with a minimization of the other properties. Through these analyses, each of the commonly discussed parameters (sterics, π-π system interactions, and sulfur donation strength) can be independently examined with lesser contributions from the other effects.

Interestingly, the AL alkyl thiol protected AuNMs can be converted to other AuNMs through the use of either AR thiol or BU *tert-*butyl thiol ligands. While both of these ligands do give different core sizes, the ready conversion of the primary alkyl thiol protected AuNMs by these ligands suggests pKa and π-π interaction are not as strong in stabilizing the AuNMs as compared to ligand sterics. Importantly, the conversion of the AuNMs core-size occurs despite analyzing ligands that could also π-stack or that have similar pKa values. This conversion suggests that these factors alone do not control the AuNMs' core size and sterics plays a significant role. The critical experiments of attempting to convert AR and BU *tert-*butyl thiol protected AuNMs core-sizes back to that of the AL alkyl thiol show that the stabilization which occurs through the use of large ligands changing Au core-sizes cannot be readily reversed (Rambukwella et al., 2017b). Thus, sterics are a dominant factor in predicting the AuNMs cores size based on ligand selection.

In our previous report, we have investigated the steric ligand effect on Au_38_ system and subsequent core-size conversion to Au_30_ system indicating a clear bulky ligand effect (Rambukwella et al., 2017b). Compared to aliphatic ligands, in the class of bulky ligands, steric effect dominates over aromatic effect, thereby physicochemically different series of AuNMs are observed where sterics governs the stability of the AuNMs. The *tert*-butylthiol is a classic example of a bulky ligand (Scheme 2e and Figure S1) where, head S atom is directly bond to a tertiary carbon atom. Most widely investigated robust and thermodynamically most stable NM series with this ligand are comprised of Au_23_(S-*t*Bu)_16_ (Hesari and Workentin, 2014), and Au_30_(S-*t*Bu)_18_ (Crasto and Dass, 2013; Yang et al., 2014; Dass et al., 2016; Jones et al., 2018). Similarly, bulky adamantanethiol (S-Adm) ligand has been shown to form Au_24_(S-Adm)_16_ (Crasto et al., 2014a), Au_30_(S-Adm)_18_ (Higaki et al., 2016) and Au_21_(S-Adm)_15_ (Chen et al., 2016a; Jones et al., 2017) NMs governed by the steric ligand effect. Interestingly to date larger sizes (Au atoms > 100) of BU thiol protected NMs have not been reported. This could be due to the BU ligands hindering the growth of the AuNMs as it blocks metal atom transportation to the core as the size increases. Therefore, we believe that tuning of the synthetic protocol may be required for the synthesize of larger BU thiolate-protected AuNMs. It has been shown by Krommenhoek et al. that use of BU thiols such as adamantanethiol and cyclohexanethiol (SCy) results in smaller core sizes and narrows down the size distribution (Krommenhoek et al., 2012). In their synthesis they discovered Au_30_(S-Adm)_18_, Au_39_(S-Adm)_23_, Au_65_(SCy)_30_, and Au_67_(SCy)_30_ NMs. Interestingly for BU thiolate ligand protected NMs, more Au atoms per thiolate ligand were observed due to steric crowding at the thiolate monolayer. Also, Chen et al. have demonstrated the effect of steric hindrance on the formation of Au_130_(p-MBT)_50_, Au_104_(m-MBT)_41_, and Au_40_(o-MBT)_24_ NMs using isomeric para, meta, and ortho substituted methylbenzene thiols (MBT), respectively (Chen et al., 2015a). The authors synthesized three different NMs from the one starting precursor mixture and the results were attributed to sterics governed by the methyl group of the ligand, where the closer the methyl group to the sulfur atom in the thiol, the more steric hindrance would be. Due to the steric crowding by the bulky thiolate ligands such as *tert*-butylthiol and adamantanethiol, NMs such as Au_25_(S-AL)_18_, Au_36_(S-AR)_24_, Au_38_(S-AL)_24_, Au_144_(S-AL)_60_, or Au_279_(S-AR)_84_ cannot be synthesized, instead only a series of AuNMs governed by steric effect is being formed (Scheme 2e and Table 1).

In contrast to AL and BU ligands, the class of aromatic thiolate ligands have a direct aromatic effect due to the presence of phenyl aromatic ring immediately bond to the head S atom (Figure S1). Due to presence of aromatic rings, π electrons contribute to favorable ligand-ligand attractions which results in stabilizing the ligand shell rather than a repulsive steric effect. In contrast to the AL and BU thiolate-protected NMs, the electronic conjugation due to aromatic ligands result in bathochromic shift in optical features and thereby reduce the band gap energy of the AuNMs (Rambukwella et al., 2017a). Also, it has been discovered that contribution from phenyl ring is very critical in manifestation of metallic properties in the form of surface plasmons for Au_279_ (Sakthivel et al., 2018). Thiophenol is a classic example of aromatic thiol. TBBT thiol is found to have similar aromatic ligand effect with minimal variation of the end product except for instance reported case where, Au_133_(SR)_52_ (Nimmala et al., 2015) is reported with TBBT, but not with thiophenol. This is expected as the surface availability of the nanomolecule decreases as the size increases steric effect by *para*-tert-butyl groups becomes significant compared to smaller core-size Au_36_(SR)_24_. Similarly, it was found that physicochemical differences between thiophenol, 4-methoxybenzene thiol, 4-methylbenzene thiol, and 4-bromobenzene thiol are subtle and results in same composition of AuNMs (Nimmala and Dass, 2014; Chen et al., 2015a; Rambukwella et al., 2015; Rambukwella and Dass, 2017). Although, Au_36_(SR)_24_ NMs system was reported (Das et al., 2014) with bulky cyclopentane thiol, exclusive formation of Au_36_(SR)_24_ is dominated by electronic effect by aromatic nature of the ligand rather than steric effect. Thus, in terms of competing electronic and steric effect in the same ligand, thermodynamically stable and final composition of the product would be determined by the overall dominating factor; electronic or steric (Chen et al., 2015a). It should be noted that, in our previous work, we have shown that kinetic product Au_38_(SPh)_24_ NMs can be synthesized and isolated under controlled ligand exchange reaction conditions while core-size converts to Au_36_(SPh)_24_ if subjected to prolonged reaction (Rambukwella et al., 2017a). BU or AL thiol-protected NMs such as Au_21_(S-BU)_15_, Au_25_(S-AL)_18_, Au_30_(S-BU)_18_, Au_38_(S-AL)_24_, or Au_144_(S-AL)_60_ cannot be synthesized with aromatic thiolate ligands (Scheme 2d and Table 1).

## Ligand effect demonstrated by molecular conversion and interconversion

Ligand exchange protocols on AuNMs are a highly versatile strategy that is employed to tune the synthesis to obtain new nanomolecules that are difficult to obtain via direct Brust method (Brust et al., 1994; Hostetler et al., 1999). Ligand exchange reactions on molecular pure AuNMs allows us to investigate the influence of thiolate ligand on AuNMs' structure and to understand the fundamental aspects of ligand effect on structural selectivity (Kurashige et al., 2012; Indrasekara et al., 2014; Carducci et al., 2015; Higaki et al., 2016). It has been demonstrated by many, that a distinct AuNMs can be converted to a new one via ligand exchange in the presence of a physicochemically different ligand (Kamei et al., 2011; Zeng et al., 2013; Nimmala et al., 2014a, 2015; Bootharaju et al., 2015; Rambukwella et al., 2017b).

**Scheme 3:**
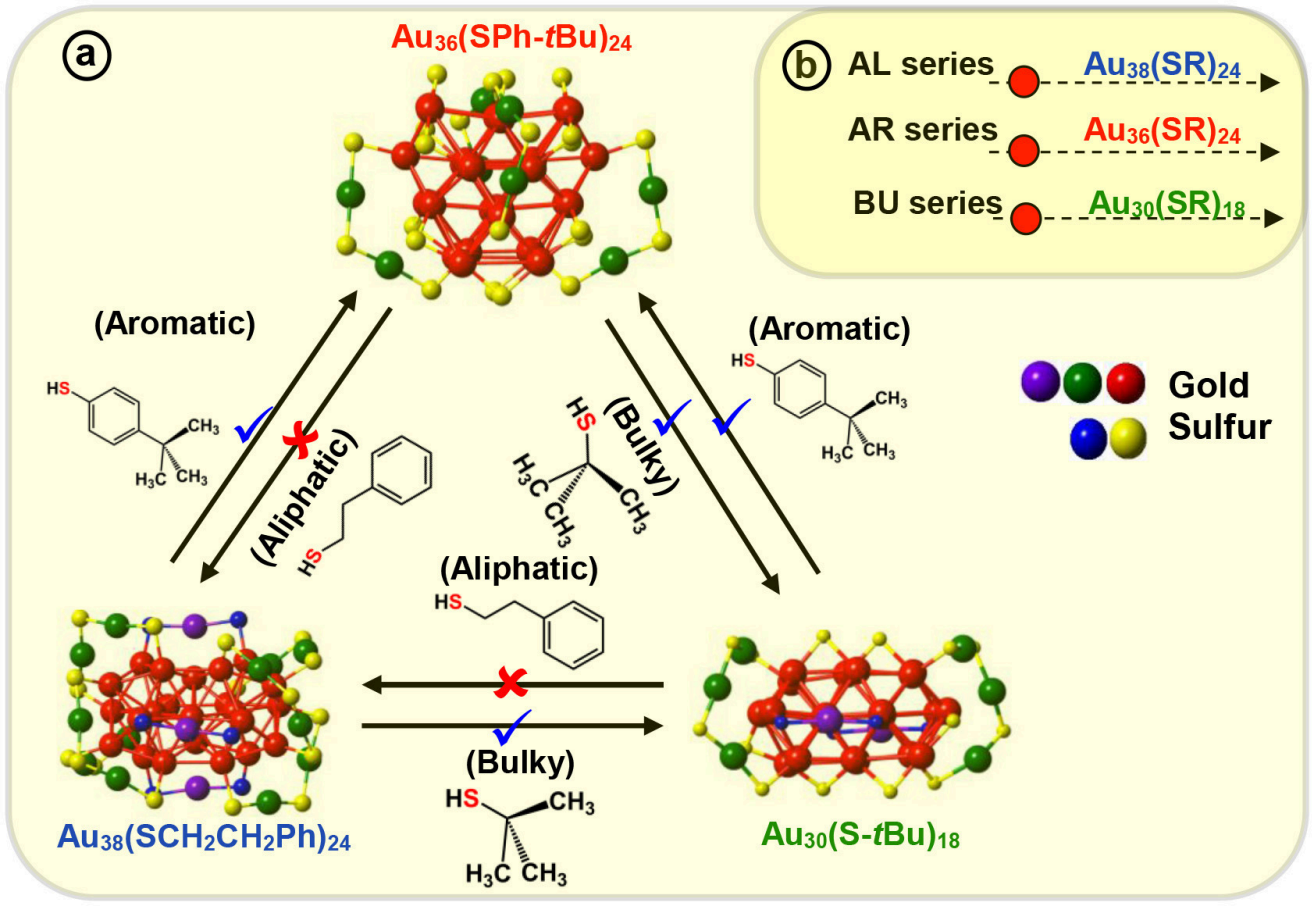
**(a)** Molecular interconversion between Au_38_(SCH_2_CH_2_Ph)_24_, Au_36_(SPh-*t*Bu)_24_, and Au_30_(S-*t*Bu)_18_ NMs. Red (✘) indicates interconversion that is restricted or not observed whereas blue (✔) indicates possible and observed interconversions. **(b)** Three series of aliphatic (AL), aromatic (AR), and, bulky (BU) NMs illustrating most stable NMs in each series, Au_38_(SCH_2_CH_2_Ph)_24_, Au_36_(SPh-*t*Bu)_24_, and Au_30_(S-*t*Bu)_18_ respectively.

For the first time, we have demonstrated the ligand induced interconversion between Au_30_(S-tBu)_18_ and Au_36_(SPhX)_24_ NMs (where, X = –H, –tBu, Scheme 3) (Dass et al., 2017). The two AuNMs, Au_30_(S-tBu)_18_, and Au_36_(SPhX)_24_ have interpenetrating-cuboctahedral Au_20_ and Au_28_ core structures, respectively and completely different staple motifs (Scheme 3). This discovery leads to *a valuable insight into the inherent nature of ligand structure dependency on atomic structure* of thiolate protected AuNMs. The experiment was carried out on molecular pure starting materials, Au_30_(S-tBu)_18_ and Au_36_(SPh-X)_24_ NMs, which were reacted with TBBT and *tert*-butyl thiol at elevated temperature, respectively (Scheme 3). The results demonstrated that when Au_30_(S-tBu)_18_ is treated with aromatic thiophenol or TBBT, the core converts to the preferred Au_36_(SPhX)_24_ structure, as dictated by the exchanging ligand. Likewise, when Au_36_(SPhX)_24_ is treated with the bulky *tert*-butyl thiol, it converts to the preferred and most stable structure Au_30_(S-tBu)_18_ NMs. It should be underlined that the interconversion of each AuNMs completes with the respective thiolate ligand retaining its original physicochemical properties, i.e., Au_36_ and Au_30_ formed upon interconversion reaction possess their intrinsic properties unaltered. Au_38_(SCH_2_CH_2_Ph)_24_, a similar core sized AuNM was reported to undergo transformation to Au_30_(S-tBu)_18_ (Rambukwella et al., 2017b) and Au_36_(SPh-tBu)_24_ (Zeng et al., 2013) NMs when etched with *tert*-butyl thiol and TBBT, respectively. Interestingly, when the products were etched with AL ligand it was found that Au_30_ or Au_36_ was not completely converted back to Au_38_ system retaining its original physicochemical properties. Instead a mixture of NMs composing Au_38_(SCH_2_CH_2_Ph)_24_, Au_38_(SCH_2_CH_2_Ph)_26_ and other small NMs was observed. This could be due to the difference in core structures, where Au_38_ has an icosahedron core and Au_36_ and Au_30_ has cuboctahedron core structures. Also, it could be attributed to the description on Scheme 3 revealing the interconversion being allowed between bulky and aromatic thiolate protected AuNMs and not with the primary alkyl thiolate ligands. Therefore, complete atomic rearrangement is “*forbidden*” in the case of converting Au_30_ or Au_36_ to large Au_38_ system. Thus, these results demonstrate that atomic structure and metal-ligand interface of NMs can be tuned with AL, AR and BU ligands.

To provide theoretical information on the thermodynamic stability of Au_38_(SCH_2_CH_2_Ph)_24_, Au_36_(SPh)_24_, and Au_30_(StBu)_18_ nanomolecules, we compared their energetics using analysis tools proposed in previous work (Reimers et al., 2010; Jung et al., 2012; Crasto et al., 2014a; Nimmala et al., 2015), in particular:

(1) energy decomposition (fragmentation) (Crasto et al., 2014a), and (2) system comparison (Jung et al., 2012; Nimmala et al., 2015) procedures.

### Energy decomposition (fragmentation) analysis

The first point to be noted when comparing the Au_38_(SCH_2_CH_2_Ph)_24_, Au_36_(SPh)_24_, and Au_30_(StBu)_18_ series is that the strength of S-Au bonds is different in aliphatic vs. aromatic thiols, and, as previously pointed out in the literature (Jung et al., 2012), it parallels the strength of S-H bonds. Specifically, if we compare the bond strength given by the process: HSR ➔ H + SR, we find that the reaction energy is nearly the same for H-StBu and H-SCH_2_CH_2_Ph: 4.86 eV and 4.87 eV, respectively, which should correspond to a similar covalent bonding of these ligands to gold, whereas it is different in H-SPh: 4.50 eV, i.e., smaller by 0.36–0.37 eV. As we discuss below, the different strength of the Au-S bond is one important factor determining the differences in chemical behavior of aliphatic vs. aromatic ligands, other important factors being steric effects associated with the larger hindrance of –StBu with respect to –SCH_2_CH_2_Ph or electronic effects such as conjugation and resonance associated with thiols exhibiting aromatic rings directly bound to sulfur such as –SPh-R.

A second point is the question of how to compare nanomolecules with a different stoichiometry – Au_N_(SR)_M_. Clearly, their energetics must be properly normalized for such a comparison to be meaningful. Here we use the number of ligands, M, as a normalization factor, so that all quantities here reported should be intended as per ligand (absolute energy values used to calculate fragmentation and charging energies are reported in Table S1 of the SI).

**Figure 2:**
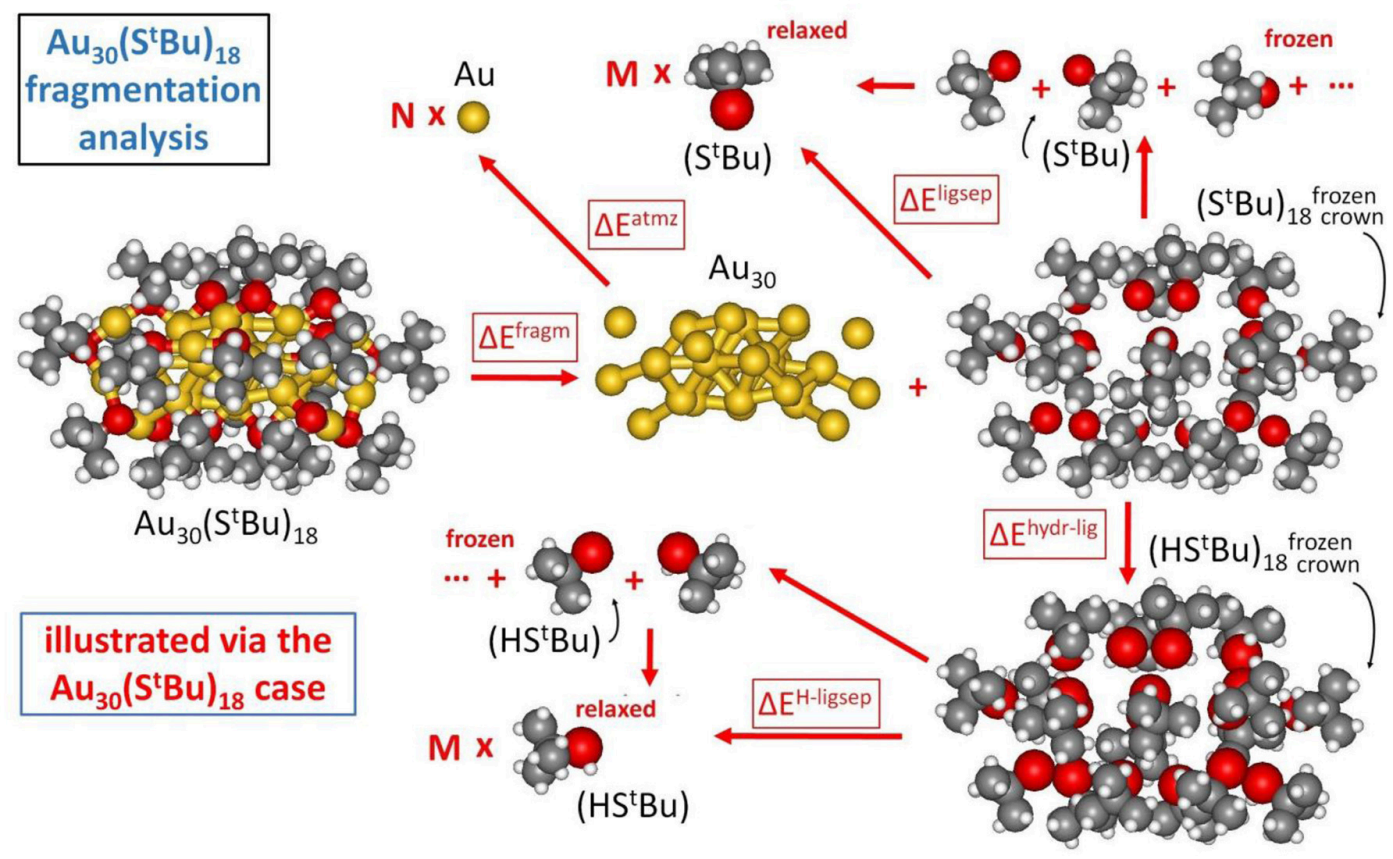
Fragmentation analysis of the energy of Au nanomolecules, illustrated for definitiveness in the case of Au_30_(S-tBu)_18_.

In our approach, the formation energy of a Au_N_(SR)_M_ nanomolecule is partitioned into three components (Crasto et al., 2014a), as schematically illustrated in Figure 2:

1. cluster fragmentation – ΔE_fragm_
2. metal atomization – ΔE_atmz_
3. ligand separation – ΔE_ligsep_

so that the formation energy of a Au_N_(SR)_M_ cluster from N, Au atoms and M, SR thiyl radicals – E_form_ (at T = 0 K and neglecting vibrational and entropic contributions) can be expressed as: E_form_ = ΔE_fragm_ + ΔE_atmz_ + ΔE_ligsep_.

**Figure 3:**
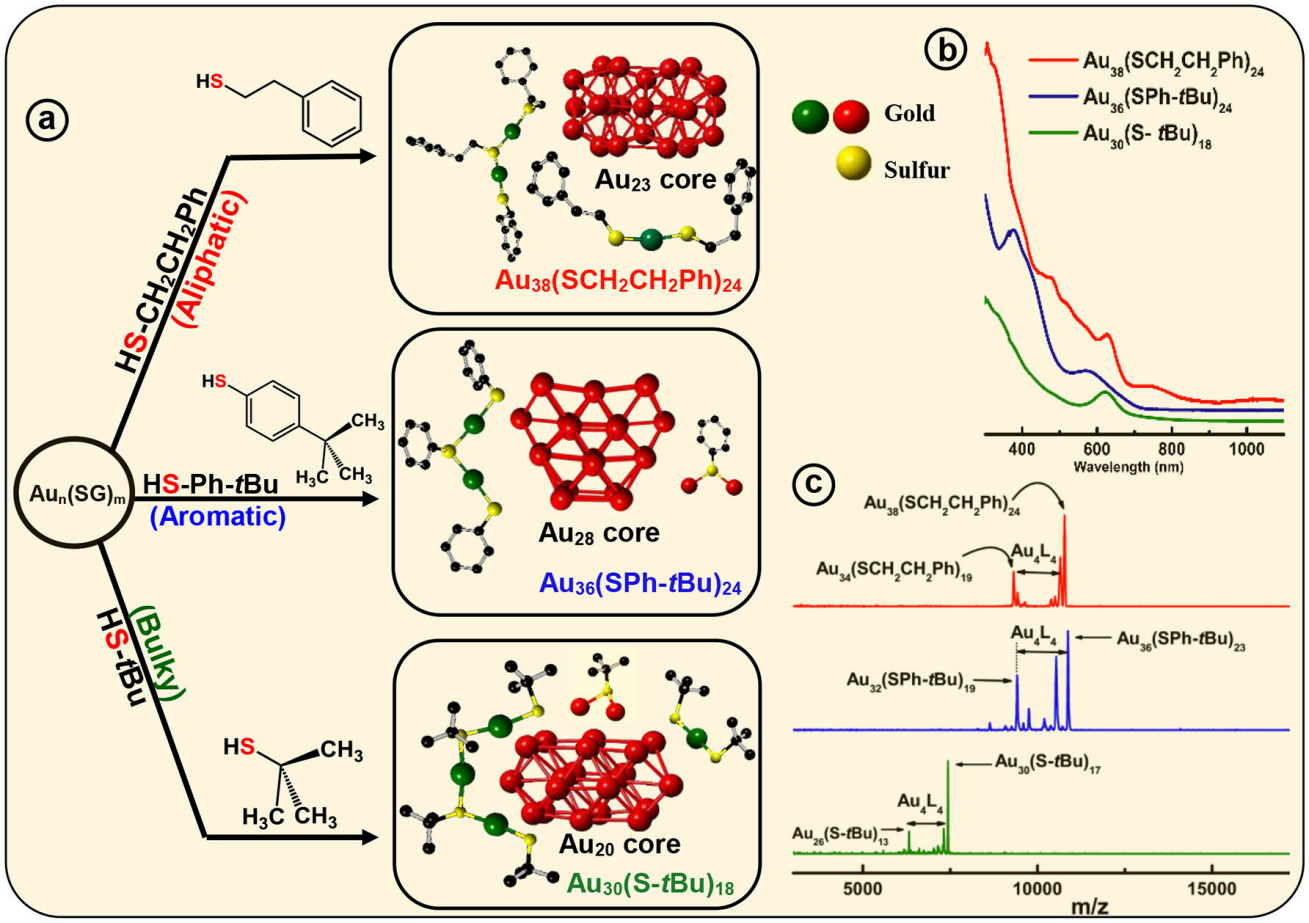
Ligand effect on the common Au_n_(SG)_m_ crude nanocluster mixture. **(a)** Upon thermochemical treatment on Au_n_(SG)_m_ kinetic product, with aliphatic ligand HSCH_2_CH_2_Ph, aromatic ligand HSPh-*t*Bu and bulky ligand HS-*t*Bu produce, monodisperse Au_38_(SCH_2_CH_2_Ph)_24_, Au_36_(SPh-*t*Bu)_24_, and Au_30_(S-*t*Bu)_18_ NMs, respectively. Corresponding assembly of crystal structures are shown to illustrate different core geometry and ligand environments (red - core Au, green-dimeric staple Au and monomeric staple Au, yellow–staple S and bridging S). **(b)** Corresponding UV-Vis-NIR absorption spectra illustrate the signature absorbance features unique to each individual NMs. **(c)** MALDI-MS of the core-size converted final products namely, Au_38_(SCH_2_CH_2_Ph)_24_, Au_36_(SPh-*t*Bu)_24_ and Au_30_(S-*t*Bu)_18_ NMs. Analytes were intentionally fragmented to demonstrate Au_4_(SR)_4_ loss, that is prominent in all three NMs.

The first component corresponds to fragmentation of the nanomolecule into a metal cluster and a “crown” of ligands, with the reaction energy normalized to the number of ligands, M, as anticipated above:

(t1) $$\begin{array}{l} {\text{Au}}_{38}{\left({\text{SCH}}_{2}{\text{CH}}_{2}\text{Ph}\right)}_{24}\;➔{\text{Au}}_{38}\;+\\ \;{\left({\text{SCH}}_{2}{\text{CH}}_{2}\text{Ph}\right)}_{24}^{\text{crown}}\\ \;\Delta {\text{E}}_{\text{fragm}/\text{M}}=3.440\text{ eV} \end{array}$$

(t2) $$\begin{array}{l} {\text{Au}}_{36}{\left(\text{SPh}\right)}_{24}\;➔{\text{Au}}_{36}+{\left(\text{SPh}\right)}_{24}^{\text{crown}}\\ \;\Delta {\text{E}}_{\text{fragm}/\text{M}}=3.095\text{ eV} \end{array}$$

(t3) $$\begin{array}{l} {\text{Au}}_{30}{\left(\text{StBu}\right)}_{18}\;➔{\text{Au}}_{30}\;+\;{\left(\text{StBu}\right)}_{18}^{\text{crown}}\\ \;\Delta {\text{E}}_{\text{fragm}/\text{M}}=3.532\text{ eV} \end{array}$$

where the coordinates of the Au_38_, Au_36_, Au_30_, ${\left(\text{SR}\right)}_{\text{24}}^{\text{crown}}$ and ${\left(\text{SR}\right)}_{\text{18}}^{\text{crown}}$ fragments in the right-hand-side of the equations are frozen in their interacting configurations, ΔE_fragm_ is the reaction energy and is reported per ligand (ΔE_fragm/M_). Without entering into a finer analysis considering the difference between ligand detachment from monomeric, dimeric, and trimeric staples (see Figure 3a, Table S1, and Figure S2), it can be noted from Equations (t1–t3) that Au_36_(SPh)_24_ has the smallest fragmentation energy, due to its aromatic nature discussed above (Jung et al., 2012; Crasto et al., 2014a): the difference in ΔE_fragm/M_ between (t1) and (t2) is indeed close to 0.36–0.37 eV, while the larger value of ΔE_fragm/M_ for Au_30_(StBu)_18_ can be explained by its sparser ligand density at the surface which increases the strength of Au-S bonds.

The second component is the atomization energy of the metal cluster:

(t4) $$\begin{array}{l} {\text{Au}}_{38}\left[{\text{fromAu}}_{38}{\left({\text{SCH}}_{2}{\text{CH}}_{2}\text{Ph}\right)}_{24}\right]\;➔38\text{ Au}\\ \;\Delta {\text{E}}_{\text{atmz}/\text{M}}=3.257\text{ eV} \end{array}$$

(t5) $$\begin{array}{l} {\text{Au}}_{36}\left[{\text{fromAu}}_{36}{\left(\text{SPh}\right)}_{24}\right]\;➔36\text{ Au}\\ \;\Delta {\text{E}}_{\text{atmz}/\text{M}}=3.087\text{ eV} \end{array}$$

(t6) $$\begin{array}{l} {\text{Au}}_{30}\left[{\text{fromAu}}_{30}{\left(\text{StBu}\right)}_{18}\right]\;➔30\text{ Au}\\ \;\Delta {\text{E}}_{\text{atmz}/\text{M}}=3.440\text{ eV} \end{array}$$

In this case it is worthwhile reporting also the atomization energies normalized by the number of Au atoms: ΔE_atmz/N_[Au_38_] = 2.057 eV, ΔE_atmz/N_[Au_36_] = 2.058 eV, ΔE_atmz/N_[Au_30_] = 2.064 eV. By comparing the resulting energy values, we find again that Au_30_(StBu)_18_ seems to be more stable than Au_38_(SCH_2_CH_2_Ph)_24_, because ΔE_atmz/N_[Au_30_] is larger than ΔE_atmz/N_[Au_38_] in absolute value, which likely is even stronger considering that Au_38_ is larger than Au_30_ and should thus exhibit a larger atomization energy (usually increasing with increasing cluster size). By defining first neighbors of a given Au atom as all Au atoms within 3.2 Å, which is the inflection point in the plot of Au-Au distances, we can monitor Au-Au first-neighbor distances, finding that Au_38_(SCH_2_CH_2_Ph)_24_ exhibits an average Au-Au first-neighbor distance of 2.95 Å, that is larger than in Au_30_(StBu)_18_, where it amounts to 2.91 Å, thus explaining the lesser stability of the Au_38_ metal cluster.

The last component of our energy fragmentation analysis corresponds to the decomposition of the crown or shell of ligands into separated thiyl radicals (we allow the radicals to fully relax in this analysis):

(t7) $$\begin{array}{l} {\left({\text{SCH}}_{2}{\text{CH}}_{2}\text{Ph}\right)}_{24}^{\text{crown}}\;➔24{\left({\text{SCH}}_{2}{\text{CH}}_{2}\text{Ph}\right)}_{\text{relax}}\\ \;\Delta {\text{E}}_{\text{ligsep}/\text{M}}=1.137\text{ eV } \end{array}$$

(t8) $$\begin{array}{l} {\left(\text{SPh}\right)}_{24}^{\text{crown}}\;➔24{\left(\text{SPh}\right)}_{\text{relax}}\\ \;\Delta {\text{E}}_{\text{ligsep}/\text{M}}=0.158\text{ eV } \end{array}$$

(t9) $$\begin{array}{l} {\left(\text{StBu}\right)}_{18}^{\text{crown}}\;➔24{\left(\text{StBu}\right)}_{\text{relax}}\\ \;\Delta {\text{E}}_{\text{ligsep}/\text{M}}=0.179\text{ eV } \end{array}$$

The similar values for ${\left(\text{StBu}\right)}_{\text{18}}^{\text{crown}}$ and ${\left(\text{SPh}\right)}_{\text{24}}^{\text{crown}}$ can be noted, despite their different origin in π-π and T-stackings interactions among phenyl rings (Nimmala et al., 2015) in the aromatic case with respect to CH/CH dispersion interactions (Fortunelli and Selmi, 1994) in the bulky aliphatic case. However, the most dramatic difference is associated with the huge value of ΔE_ligsep/M_ for Au_38_(SCH_2_CH_2_Ph)_24_. To single out the physical origin of this striking difference we need to make a further analysis and distinguish two terms in the ΔE_ligsep_ separation energy: the energy of the S-S bonds among the under-coordinated sulfur atoms of the thiyls (some of the sulfur atoms in the interacting configuration are at close distance) and dispersion/repulsion interactions among the organic residues. To separately estimate these contributions, we first hydrogenate the sulfur atoms of the thiyl radicals in the ligand crown, relaxing the geometry of the added H atoms while keeping all other atoms frozen (ΔE^hyrd−lig^), and then calculate the separation energy of the thus formed thiol ligand shell into relaxed and separated thiols (ΔE^H−ligsep^), as pictorially illustrated in Figure 2:

(t10) $$\begin{array}{l} {\left({\text{SCH}}_{2}{\text{CH}}_{2}\text{Ph}\right)}_{24^{\text{crown}}}+\;24\text{H}➔{\left({\text{HSCH}}_{2}{\text{CH}}_{2}\text{Ph}\right)}^{\text{crown}}\\ \;\Delta {\text{E}}_{\text{hyrd}-\text{lig}/\text{M}}=4.847\text{ eV } \end{array}$$

(t11) $$\begin{array}{l} {\left(\text{SPh}\right)}_{24}^{\text{crown}}+24\text{H}➔{\left(\text{HSPh}\right)}_{24}^{\text{crown}}\\ \;\Delta {\text{E}}_{\text{hyrd}-\text{lig}/\text{M}}=4.597\text{ eV} \end{array}$$

(t12) $$\begin{array}{l} {\left(\text{StBu}\right)}_{18}^{\text{crown}}+18\text{H}➔{\left(\text{HStBu}\right)}_{18}^{\text{crown}}\\ \;\Delta {\text{E}}_{\text{hyrd}-\text{lig}/\text{M}}=4.861\text{ eV} \end{array}$$

and

(t13) $$\begin{array}{l} {\left({\text{HSCH}}_{2}{\text{CH}}_{2}\text{Ph}\right)}_{24}^{\text{crown}}\;➔24{\left({\text{HSCH}}_{2}{\text{CH}}_{2}\text{Ph}\right)}_{\text{relax}}\\ \;\Delta {\text{E}}_{\text{H}-\text{ligsep}/\text{M}}=1.109\text{eV} \end{array}$$

(t14) $$\begin{array}{l} {\left(\text{HSPh}\right)}_{24}^{\text{crown}}\;➔24{\left(\text{HSPh}\right)}_{\text{relax}}\\ \;\Delta {\text{E}}_{\text{H}-\text{ligsep}/\text{M}}=0.253\text{eV} \end{array}$$

(t15) $$\begin{array}{l} {\left(\text{HStBu}\right)}_{18}^{\text{crown}}\;➔24{\left(\text{HStBu}\right)}_{\text{relax}}\\ \;\Delta {\text{E}}_{\text{H}-\text{ligsep}/\text{M}}=0.178\text{eV} \end{array}$$

ΔE_hyrd−lig/M_ can be compared with the strength of the S-H bond that is 4.875 eV in H-SCH_2_CH_2_Ph, 4.503 eV in H-SPh, and 4.863 eV in H-StBu, respectively: for (StBu)_18_ it is nearly identical, whereas for (SPh)_24_ and (SCH_2_CH_2_Ph)_24_ it differs by 0.094 and 0.028 eV, respectively. Analogously, the separation energy of hydrogenated ligands for Au_30_(StBu)_18_ is nearly identical to that of the thiyl radical shell, this quantity is also close for Au_38_(SCH_2_CH_2_Ph)_24_ (1.109 vs. 1.137 eV), whereas for Au_36_(SPh)_24_ the value of 0.253 eV is somewhat larger than 0.159 eV for thiyl radicals, due to the fact that residual S-S bonds are weakened by conjugation. Notably, this proves that ligand-ligand interactions between phenyl rings (Nimmala et al., 2015), in which the major role is played by dispersion interactions among the organic residues, especially π-π and T-stackings interactions, account for the huge value of ΔE_ligsep/M_ in Au_38_(SCH_2_CH_2_Ph)_24_, and should thus be kept in mind when rationalizing the reasons of the experimentally observed thermodynamic stability of this compound. In particular, as discussed in Ref. (Crasto et al., 2014a), this stabilization will depend in a decisive way on the cluster environment, i.e., the solvent or the crystal, and will also depend on effects of configurational entropy, important for such floppy systems (but here provisionally neglected). Considering solvated species for example, we can expect that Au_38_(SCH_2_CH_2_Ph)_24_ will adopt a “brush” conformation in more “wetting” (more strongly interacting) solvents, such as e.g., benzene, and a “mushroom” conformation in less “wetting” solvents such as chloroform (de Gennes, 1980), possibly leading to its lesser stability in such media.

Finally, we note that for specific compounds here investigated we do not need to distinguish dispersion and repulsion interactions among the organic residues, i.e., attractive dispersion interactions from steric repulsion, due to the fact that steric repulsion is small in these species. It is however possible to make this distinction approximately quantitative as discussed in Ref. (Rambukwella et al., 2015) via a system comparison approach of the type reviewed in the next sub-section, i.e., by transforming into and comparing with non-sterically hindered species via ligand exchange and matching the resulting energetics.

### System comparison analysis and chemical potentials

To shed further light on interconversion processes experimentally investigated, it is useful to calculate direct energy balances among different mono-layer protected cluster species. In a previous analysis (Crasto et al., 2014a) we analyzed the energetics of processes such as incremental formation or addition:

(t16) $$\begin{array}{l} {\text{Au}}_{\text{N}}{\left(\text{SR}\right)}_{\text{M}}+\text{Au }➔{\text{Au}}_{\text{N+1}}{\left(\text{SR}\right)}_{\text{M}}\\ \text{ metal addition} \end{array}$$

(t17) $$\begin{array}{l} {\text{Au}}_{\text{N}}{\left(\text{SR}\right)}_{\text{M}}+\text{HSR }➔{\text{Au}}_{\text{N}}{\left(\text{SR}\right)}_{\text{M}+1}+½{\text{H}}_{2}\\ \text{ ligand addition} \end{array}$$

(t18) $$\begin{array}{l} {\text{Au}}_{\text{N}}{\left(\text{SR}\right)}_{\text{M}}+{\text{e}}^{-}\;➔{\text{Au}}_{\text{N}}{\left(\text{SR}\right)}_{\text{M}}^{-}\\ \text{ electron affinity} \end{array}$$

In this analysis one needs values for the chemical potentials of an Au atom, μ(Au), a ligand thiol, μ (HSR), and an electron, μ (e^*-*^), in addition to QM total energies. In the present context we won't use the electron chemical potential (t18) as all species here considered are neutral, and for an easier read we will replace (t16, t17) with a ligand exchange and cluster inter-conversion processes:

(t19) $$\begin{array}{l} {\text{Au}}_{{\text{N}}^{\prime }}{\left({\text{SR}}^{\prime }\right)}_{{\text{M}}^{\prime }}+{\text{M}}^{\prime }\left(\text{HSR}\right)➔{\text{Au}}_{{\text{N}}^{\prime }}{\left(\text{SR}\right)}_{{\text{M}}^{\prime }}+{\text{M}}^{\prime }\left({\text{HSR}}^{\prime }\right)\\ \text{ ligand exchange} \end{array}$$

(t20) $$\begin{array}{l} {\text{NAu}}_{{\text{N}}^{\prime }}{\left(\text{SR}\right)}_{{\text{M}}^{\prime }}+{\text{MN}}^{\prime }\left(\text{SR}\right)➔{\text{N}}^{\prime }{\text{Au}}_{\text{N}}{\left(\text{SR}\right)}_{\text{M}}+{\text{NM}}^{\prime }\left(\text{SR}\right)\\ \text{ cluster interconversion} \end{array}$$

where we denote the reaction energy of the ligand exchange process (t19) as ΔE_lig−exch_, and that of the cluster interconversion process (t20) as ΔE_int−conv_. It should be noted that, while Au_N′_(SR')_M′_ and Au_N_(SR)_M_ are experimentally determined compounds, Au_N′_(SR)_M′_ intermediate species are not necessarily so, and their energetics have here been derived via simulations as described in the computational details.

Without analyzing all possible interconversion processes, let us focus on the most salient information that can be derived using Equations (t19, t20) in addition to that already singled out in the previous sub-section. First, it is instructive to system-compare via ligand exchange Au_38_(SCH_2_CH_2_Ph)_24_ and Au_38_(SCH_3_)_24_. In detail, the reaction energy for ligand exchange ΔE_lig−exch_ for “Au_38_(SCH_2_CH_2_Ph)_24_ ➔ Au_38_(SCH_3_)_24_” amounts to 24.285 eV, and is perfectly matched by the difference in ligand separation energy, ΔE_ligsep_, between ${\left({\text{SCH}}_{\text{2}}{\text{CH}}_{\text{2}}\text{Ph}\right)}_{24}^{\text{crown}}$ [from-Au_38_(SCH_2_CH_2_Ph)_24_] and ${\left({\text{SCH}}_{3}\right)}_{24}^{\text{crown}}$[from-Au_38_(SCH_3_)_24_] = 24.276 eV. Not surprisingly, the energy difference between these two chemically similar species is *exclusively due to ligand-ligand interactions*.

Less obvious is that the ligand exchange reaction energy ΔE_lig−exch_ for “Au_38_(SCH_2_CH_2_Ph)_24_ ➔ Au_38_(SPh)_24_” amounts to 16.498 eV and is partially albeit largely matched by the difference in ligand separation energy, ΔE_ligsep_, between ${\left({\text{SCH}}_{\text{2}}{\text{CH}}_{\text{2}}\text{Ph}\right)}_{24}^{\text{crown}}$[from-Au_38_(SCH_2_CH_2_Ph)_24_] and ${\left(\text{SPh}\right)}_{24}^{\text{crown}}$[from-Au_38_(SPh)_24_] = 15.809 eV. In other words, it turns out that the ligand exchange process “Au_38_(SCH_2_CH_2_Ph)_24_ ➔ Au_38_(SPh)_24_” is also dominated by ligand-ligand interactions plus an additional stabilization of ≈0.7 eV. This is in tune with the fact that Au_38_(SPh)_24_ can indeed be synthesized under appropriate experimental conditions (Rambukwella et al., 2017b): a stabilization of ≈0.7 eV can in fact be overcome by playing with the chemical potentials μ(HSCH_2_CH_2_Ph) and μ(HSPh) (i.e., changing temperature and reactant concentrations).

In contrast, the ligand exchange reaction energy ΔE_lig−exch_ for “Au_30_(SPh)_18_ ➔ Au_30_(StBu)_18_” amounts to 1.185 eV and is over-matched by the difference in ligand separation energy, ΔE_ligsep_, between ${\left(\text{SPh}\right)}_{18}^{\text{crown}}$[from-Au_30_(SPh)_18_] and ${\left(\text{StBu}\right)}_{18}^{\text{crown}}$[from-Au_30_(StBu)_18_] = 2.162 eV. In other words, Au_30_(SR)_18_ prefers to be in the bulky form, Au_30_(StBu)_18_ with respect to the aromatic form Au_30_(SPh)_18_ by ≈1.0 eV (neglecting ligand-ligand interaction effects). This suggests that Au_30_(SPh)_18_ could also be synthesized under carefully controlled conditions by playing with the chemical potentials μ(HSPh) and μ(HStBu).

As an example of cluster inter-conversion process, we take “Au_38_(SPh)_24_ ➔ Au_36_(SPh)_24_”:

(t21) $$\begin{array}{l} 36{\text{Au}}_{38}{\left(\text{SR}\right)}_{24}24\text{x38}\left(\text{SPh}\right)➔38{\text{Au}}_{24}{\left(\text{SPh}\right)}_{24}\\ \;+24\text{x36}\left(\text{SPh}\right) \end{array}$$

or, equivalently,

(t22) $$36/24{\text{Au}}_{38}{\left(\text{SR}\right)}_{24}+2\left(\text{SPh}\right)➔38/24{\text{Au}}_{24}{\left(\text{SPh}\right)}_{24}$$

The corresponding energy ΔE_int−conv/M_ normalized to the number of ligands (*M* = 24) amounts to 2.983 eV. To further quantify this value, we need the chemical potential of SPh, μ(SPh), which we roughly estimate as the energy of the reaction:

(t23) $$\begin{array}{ll} \text{HSPh}➔½{\text{H}}_{2}+\text{SR} & \Delta \text{E}=1.320\text{eV} \end{array}$$

Thus, obtaining a total contribution due to μ(SPh) = 2 ΔE = 2.640 eV, 0.343 eV smaller than ΔE_int−conv/M_. In other words, Au_36_(SPh)_24_ turns out to be more stable than Au_38_(SPh)_24_ by 0.343 eV, but not quite enough as to recover the extra stabilization of Au_38_(SCH_2_CH_2_Ph)_24_, which we evaluated above as ≈0.7 eV. This can be interpreted in the sense that the structure of Au_38_(SCH_2_CH_2_Ph)_24_ is indeed particularly apt to favor SCH_2_CH_2_Ph-SCH_2_CH_2_Ph interactions.

The picture drawn from this total energy analysis seems in tune with experiment and able to provide further insight. What we believe is most important is the foundation of a quantitative basis to dissect and predict the thermodynamic stability of mono-layer protected clusters. From this analysis the importance of ligand-ligand interactions clearly stands out as a determining factor, especially for species with complex ligands such as Au_38_(SCH_2_CH_2_Ph)_24_ which, according to our analysis, a substantial part of their stability is due to dispersive interactions, so that they may be expected to adopt “mushroom” conformations (de Gennes, 1980) in “non-wetting” solvents and thus be more stable in such media due to a “self-solvation” mechanism.

Clearly, we must point out that we focused here on electronic energies at equilibrium and zero temperature, i.e., we have here neglected vibrational and entropic contributions and kinetic effects. However such effects have been shown to produce important effects for some nanomolecules (Nimmala et al., 2015), and will therefore be the subject of future studies.

Finally, to make connections with electrochemical applications of these systems (Antonello and Maran, 2017) and also to extract useful quantities such as Mulliken electronegativity (Pauling, 1960) and chemical hardness (Pearson, 2005) which are commonly utilized to make semi-quantitative predictions of chemical propensity, we report vertical ionization potential (IP) and electron affinity (EA) of the nanomolecules here investigated. IP is the energy needed to extract an electron from the cluster, while EA is the energy gained in adding an electron to the cluster, respectively, while keeping the geometry frozen at that optimized for the neutral species. The ionization potentials of Au_38_(SCH_2_CH_2_Ph)_24_, Au_36_(SPh)_24_, and Au_30_(StBu)_18_ nanomolecules are: 4.72 eV, 5.44 eV, 5.06 eV, respectively, while the electron affinities are: 2.29 eV, 2.17 eV, 1.75 eV, respectively. Interestingly, the chemical hardness [(IP-EA)/2] follow the order: 1.655 eV [Au_30_(StBu)_18_] ≈ 1.635 eV [Au_36_(SPh)_24_] > 1.215 eV [Au_38_(SCH_2_CH_2_Ph)_24_], whereas for the Mulliken electronegativity [(IP+EA)/2] the order is: 3.805 eV [Au_36_(SPh)_24_] > 3.505 eV [Au_38_(SCH_2_CH_2_Ph)_24_] > 3.405 eV [Au_30_(StBu)_18_], indicating that Au_36_(SPh)_24_] most easily receives an electron from the environment (because of delocalization on phenyl rings), whereas Au_38_(SCH_2_CH_2_Ph)_24_ is thermodynamically (although probably not kinetically) and chemically the most reactive species.

**Scheme 4:**
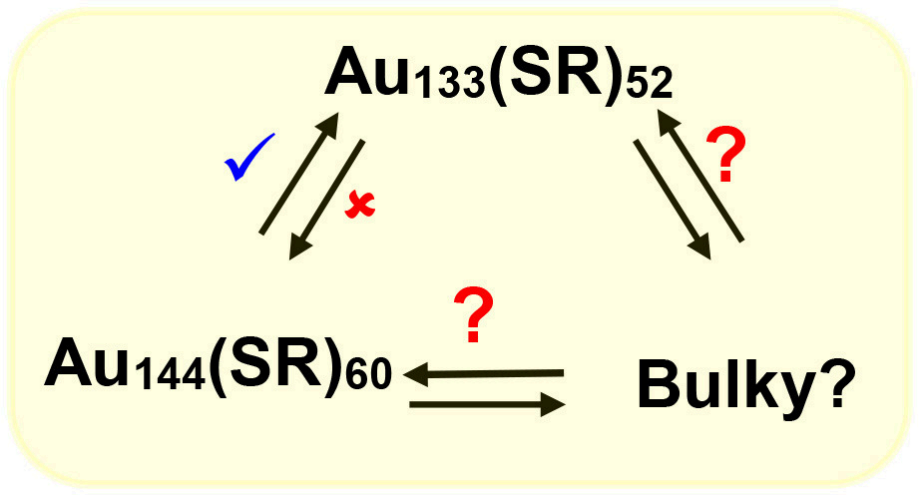
Potential molecular interconversion cycle between similar core-size Au_144_(SCH_2_CH_2_Ph)_60_, Au_133_(SPh-*t*Bu)_52_, and bulky Aun(S-*t*Bu)y NMs. Red (✘) indicates conversion that is restricted or not observed whereas blue (✔) indicates observed conversion and red (?) indicates possible or to be explored conversions.

We have shown that with aromatic ligands Au_144_(SCH_2_CH_2_Ph)_60_ core-size converts to Au_133_(SPh-*t*Bu)_52_ (Nimmala et al., 2015) and our hypothesis is that similar interconverting cycle of NMs (Scheme 4) should exist at this size regime (1.6–1.7 nm). To date NMs with bulky thiolate ligands in this size regime have not been reported and potentially bulky thiolate ligand-induced core-size conversion on Au_144_(SCH_2_CH_2_Ph)_60_ NMs can be used to explore this area.

## Ligand effect demonstrated by etching of a common precursor with different thiols

Core-size conversion reactions, initially reported as etching (Schaaff and Whetten, 1999), and more recently referred as, size-focusing (Jin et al., 2010), is a widely implemented post synthetic step that reduces the polydispersity of the initial product. It has been shown that Au_n_(SR)_m_ organo-soluble nanoclusters core-size converts to monodisperse Au_36_(SPh)_24_ NMs upon etching with thiophenol (Nimmala and Dass, 2011). In our recent report, motivated by ligand induced core-size conversion reactions, we have devised a new synthetic protocol to demonstrate the ligand effect of the three class of thiolate ligands (AL, AR, and BU) using a common precursor based experimental setup. Core-size conversion of the common precursor Au_x_(glutathiolate)_y_ nanoclusters (SG-glutathiolate) is induced by the physicochemical difference in the exchanging ligand (Figure 2A). Thus, the difference in steric and electronic effect between the two exchanging ligands plays a crucial role in determining the structure and properties of the NMs being synthesized, Au_38_(SCH_2_CH_2_Ph)_24_, Au_36_(SPh-tBu)_24_, and Au_30_(S-tBu)_18_. In the case of Au_38_(SCH_2_CH_2_Ph)_24_, continuous prolonged etching at 80°C for about 70 h results in core-size conversion of the higher clusters to thermodynamically stable Au_38_(SCH_2_CH_2_Ph)_24_ NMs in the AL series. In PET, aliphatic C_2_ chain –CH_2_CH_2_- links between the sulfur head and phenyl ring hinders the extended conjugation of π electron. Therefore, electronic effect of the phenyl ring is minimum and primarily the structure of the nanomolecule is governed by the aliphatic-like nature of the PET ligand. During this core-size conversion all other meta-stable nanoclusters transform to most stable Au_38_(SCH_2_CH_2_Ph)_24_ or undergoes decomposition (Figure 3c).

In contrast to the synthesis of Au_38_(SCH_2_CH_2_Ph)_24_, the etching reaction with TBBT is rapid and fast kinetics were observed as monodisperse thermodynamically most stable Au_36_(SPh-*t*Bu)_24_ NMs were formed after 18 h of etching the Au_n_(SG)_m_ crude mixture at 80°C. Fast reaction kinetics were observed possibly due to the relatively higher acidity of the HSPh-*t*Bu ligand compared to PET (pKa = 6.6 and pKa ≈ 10 respectively). In contrast to AL and BU ligands, the TBBT (HSPh-*t*Bu) ligands have a direct electronic effect due to the presence of a phenyl aromatic ring attached to head S atom. The π electron conjugation extended to the gold atomic core results in electronic effects. This is evidence by the reduction of S-C bond length in Au_36_(SPh-*t*Bu)_24_ NMs in contrast to Au_38_(SCH_2_CH_2_Ph)_24_ and Au_30_(S-*t*Bu)_18_ NMs (Table 2). In Au_36_(SPh-*t*Bu)_24_ NMs, overall 4.8% reduction in S-C average bond length can be seen with reference to aliphatic and bulky ligands. It is worth noting that, in our previous work we have observed geometric stability arising from π-π or T-stacking of the phenyl ring is also somewhat important for the stability of aromatic thiolate shell protected NMs (Rambukwella et al., 2015). This ligand-ligand interaction favors the stability of ligand shell by electronic interactions and minimizes the steric repulsions. In fact, bond strain is reflected in higher order staples in corresponding crystal structures. Au_30_(S-*t*Bu)_18_, has relatively more strained Au-S-Au bond angles mainly due to the bulky nature of the ligands. TBBT ligands being not as bulky as tert-butyl ligands, the interpenetrating cuboctahedral geometry of the Au_36_(SPh-*t*Bu)_24_ core results in less strained bonds relative to Au_38_(SCH_2_CH_2_Ph)_24_, where –CH_2_CH_2_- bridging of the PET ligands may eliminate π-π or T-stacking among phenyl rings. Therefore, the core-size transformation of the Au_n_(SG)_m_ nanocluster mixture to Au_36_(SPh-*t*Bu)_24_ is induced by the combined effect of sterics and aromaticity of the TBBT ligand.

**Table 2:** Comparison of bond distance and bond angles of the Au38(SCH_2_CH_2_Ph)24, Au36(SPh-tBu)24, and Au30(S-tBu)18 NMs.

**Parameter**	**Au_30_(S-tBu)_18_**	**Au_36_(SPh-tBu)_24_**	**Au_38_(SCH_2_CH_2_Ph)_24_**
*d**(S-C)***	**(1.848** ±**0.044) Å**	**(1.757** ±**0.032) Å**	**(1.842** ±**0.044) Å**
*d*(Au-S)	(2.325 ± 0.024) Å	(2.333 ± 0.016) Å	(2.325 ± 0.016) Å
*a*(Au-S-Au) monomeric staples	95.08°	–	93.53°
*a*(Au-S-Au) dimeric staples	–	94.28°	97.73°
*a*(Au-S-Au) trimeric staples	91.04°	–	–
**Maximum a(Au-S-Au) higher order staples**	**94.57**°	**103.42**°	**101.08**°

*Average bond length and angle are indicated by d and a, respectively. Average bond distances and bond angle values of the three NMs having drastic difference are bolded*.

Tailoring the structure of metal nanoparticles is of paramount importance to utilize them effectively in related applications. Lammerhofer et al. have reported a size independent but ligand chain length dependent ligand density phenomenon. They have observed that ligand density increases from 4.3 to 6.3 molecules nm^−2^ upon decreasing the ligand chain length from 3.52 to 0.68 nm. Recent reports show that ligand density and ligand environment affect cell targeting efficacy and cellular uptake in biomedicine. Figure 3a illustrates that, three physicochemically different ligands investigated in this study result in different metallic core structures with unique surface staple environments and optical properties (Figure 3b). The three classes of thiols (AL, AR, and BU) investigated in this study form unique combination of surface staple arrangement unique to each nanomolecule. Among them, the bulky tert-butyl thiol is the only thiol to form two trimeric staples (Au_3_(SR)_4_). In addition, the surface occupies two dimeric (Au_2_(SR)_3_) and six bridging (AuSR) staples that surrounds the Au_22_ bi-cuboctahedral gold core of the Au_30_(StBu)_18_ NMs. This could be a result of the bulkiness of the ligand modulating the metal core to be more elongated to accommodate the long trimeric staples to minimize the steric repulsions among ligands. In contrast, aromatic TBBT forms a four fused cuboctahedral Au_28_ gold core with a surface ligand shell of twelve bridging and four dimeric staples in Au_36_(SPh-*t*Bu)_24_ NMs, whereas the PET ligand forms an Au_23_ bi-icosahedral gold core with three monomeric (Au(SR)_2_) and six dimeric surface staples in the corresponding Au_38_(SCH_2_CH_2_Ph)_24_ NMs. Therefore, ligand structure is directly correlated to the structure and properties of the AuNMs, and it should be possible to tune atomic structure, metal-ligand interfaces and overall properties of nanoparticles and quantum dots in higher size regimes, simply by modifying the structure of the ligand.

## Nano-scaling law for physicochemically different thiolate protected series of AuNMs

**Figure 4:**
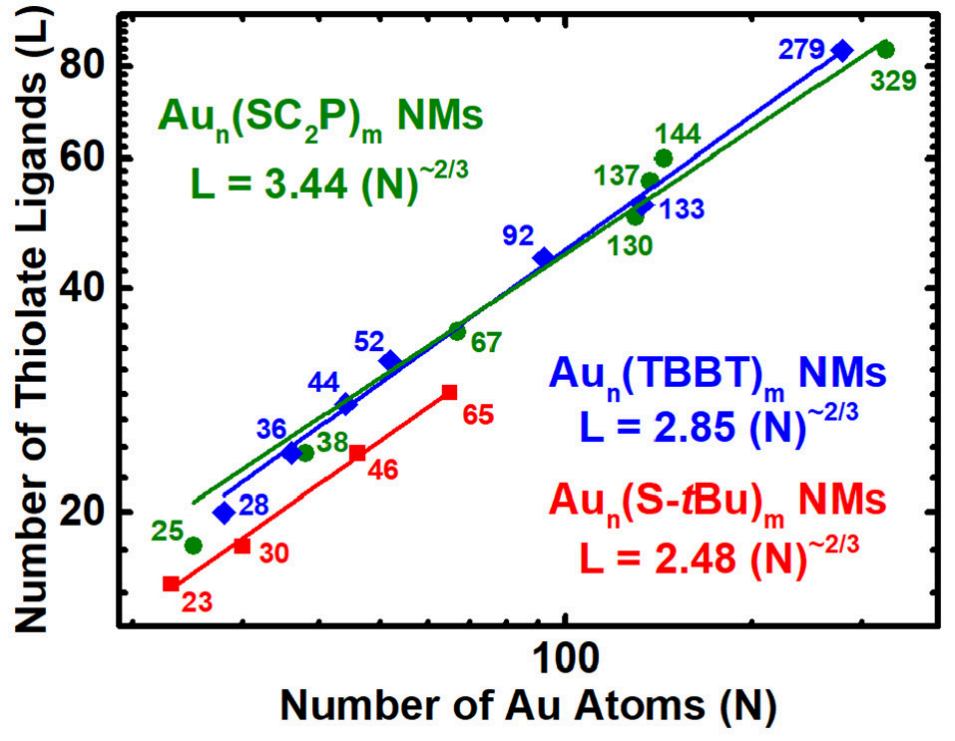
Nano-Scaling law for aliphatic, aromatic and bulky thiolated AuNMs series obtained by allometric powerfit of Log-Log plot of number of gold atoms (N) vs. thiolate ligands (L). Au_n_(SCH_2_CH_2_Ph)_m_ - olive – spheres; Au_n_(TBBT)_m_ - blue – rhombus; Au_n_(S-*t*Bu)_m_ - Red – squares. The standard errors for aliphatic, aromatic and bulky thiolated AuNMs series are slope = 0.56 ± 0.04, 0.60 ± 0.01, and 0.59 ± 0.02, respectively, and intercept = 3.44 ± 0.68, 2.85 ± 0.20, and 2.48 ± 0.23, respectively. Reduced χ^2^ = 11.48, 1.49, and 0.17, respectively; Adjusted *R*^2^ = 0.9781, 0.9970, and 0.9953, respectively. Table S2 lists the standard errors and associated values.

Common three-dimensional geometric objects such as spheres, cubes, cuboctahedras etc., are known to follow a simple Euclidean surface rule with a scaling factor of 2/3 corresponding to the surface area/volume ratio of the object. The allometric power fit of the surface area and volume of those objects provides the slope and y-intercept which relates to the scaling factor and compactness of the object. Likewise, allometric power fit of the number of gold atoms and thiolate ligands in a log-log plot has been empirically shown to provide similar insights on the AuNMs geometry and surface coverage (Dass, 2012). The ligand dictates the AuNMs core atomic structure, overall geometry and surface coverage. Distinct ligands form unique series of AuNMs and their scaling-law varies accordingly. Here we study the nano-scaling-law for three physicochemically different thiolate ligands, namely, aliphatic (PET), aromatic (TBBT) and bulky (S-*t*Bu) to determine the variability in scaling factor and compactness. Figure 4 reveals the nano-scaling for the three distinct series and Table S2 lists the standard values and errors associated with the fit. It is very interesting that all three ligand types have a very similar scaling factor of ~0.6 which is very close to the 2/3 scaling factor for regular geometric objects. However, the compactness varies evidently for each series. The compactness index for aliphatic, aromatic and bulky ligand protected AuNMs are 3.44, 2.85, and 2.48, respectively. It is intriguing that all three series are more compact than the most compact regular geometric shape (sphere) whose compactness index is 4.8 (Dass, 2012.). On the outlook, the bulky ligand protected AuNMs are more compact than aromatic and aliphatic ligands. Although the comparison between four sizes of bulky thiolated AuNMs and a large population of aliphatic and aromatic ligated AuNMs is not linear, it has to be realized that the slope trend is similar in all three AuNM series and compactness index of the bulky thiolated AuNMs might vary as the larger sizes are discovered. Overall, AuNMs follow a 2/3 scaling factor independent of the type of ligand and their compactness alone varies based on the respective ligand type.

## Conclusions

In summary, the three experimental setups demonstrated here shows that physicochemically different thiolate ligands dictate the structure, metal-ligand staple interface and induce various optical and electrochemical properties unique to individual AuNMs. The effect of the thiolate ligand can be electronic due to aromaticity or sterics or both and plays a key role in determining the thermodynamically stable structure. Attractive and steric ligand-ligand interactions are significant factors of AL, AR and BU thiolate ligands and overall dominating effect of these two factors determines the stability of the structure and properties of the AuNMs. Understanding the ligand structure dependence on atomic structure allows one to design and synthesize novel NMs. These understandings will improve the predictability of the designed synthetic protocols.

From this analysis, the importance of ligand-ligand interactions clearly stands out as a determining factor, especially for species with complex ligands such as Au_38_(SCH_2_CH_2_Ph)_24_ which, according to our computational analysis, dispersive interaction energy components make robust AuNMs in “non-wetting” solvents via a “self-solvation” mechanism.

When aromatic thiol protected AuNMs are treated with BU *tert-*butyl thiol (which have the most similar sterics of the ligands examined), a core-size change was observed. Significantly, the observation of the reverse reaction (*tert-*butyl thiol protected Au NMs convert back with the introduction of aromatic thiol ligands) proves that thiol electronics and π-π interactions does not significantly influences the conversion as pKa values of these ligands are very different and the BU *tert-*butyl thiol does not have an aryl group. Potentially, the relative steric balance struck with these ligands allows for AuNMs core-size interconversions through simple excess ligand addition driving the AuNM core-size interconversion equilibrium to a single size.

It should be noted that while these experiments do suggest that sterics is a key factor in determining AuNM core-size with large ligands giving a more stabilized Au NM structure, this does not mean that π-π interaction and electron density at the sulfur atom do not have important effects on Au NM core-size selection. However, this does predict that for these weaker effects to become evident, sterics must first be balanced between the systems being compared. This observation is in line with literature precedent, when sulfur electron density was shown to control core-size selection, the steric component was held near constant.

## Author contributions

MR contributed the experimental approach to ligand effect understanding on NMs in three aspects. NS contributed the nano-scaling law for three series of AuNMs. JD contributed the physicochemical insights of the ligands. LS and AF contributed to the computational section of this work. AD formed the concept, structure of the manuscript and supervised the manuscript preparation. All the authors made substantial, direct and intellectual contribution to the work in the manuscript preparation.

### Conflict of interest statement

The authors declare that the research was conducted in the absence of any commercial or financial relationships that could be construed as a potential conflict of interest.
