# Supplementary material for: Ligand Structure Determines Nanoparticles' Atomic Structure, Metal-Ligand Interface and Properties
Source: Front Chem. 2018 Aug 7;6:330. doi: 10.3389/fchem.2018.00330 (PMC6090168; doi:10.3389/fchem.2018.00330)
Supplement: Supplementary file 1 [file Data_Sheet_1.PDF]

Supplementary Material

Ligand Structure Determines Nanoparticles' Atomic Structure, Metal-Ligand Interface and Properties

Milan Rambukwella[1], Naga Arjun Sakthivel[1], Jared H. Delcamp[1], Luca Sementa[2], Alessandro Fortunelli[2], Amala Dass[1,*]

[1]Department of Chemistry and Biochemistry, University of Mississippi, Oxford, Mississippi 38677, United States
[2]CNR-ICCOM & IPCF, Consiglio Nazionale delle Ricerche, via Giuseppe Moruzzi 1, 56124, Pisa, Italy

Table of content

1. Figure S1. Most widely used physicochemically different thiolate ligands.
2. Computational details
3. Scheme S1. $Au_{38}(SCH_2CH_2Ph)_{24}$ synthetic protocol.
4. Scheme S2. $Au_{36}(SPh\text{-}t\text{Bu})_{24}$ synthetic protocol.
5. Scheme S3. $Au_{30}(S\text{-}t\text{Bu})_{18}$ synthetic protocol.
6. Table S1. Absolute electronic energy values for the $Au_N(SR)_M$ systems.
7. Figure S2. Schematic pictures of the $Au_N(S)_M$ skeletal frameworks.
8. Table S2. Standard values and errors associated with the nano-scaling law.
9. Cartesian coordinates of the $Au_{38}(SCH_2CH_2Ph)_{24}$, $Au_{30}(S\text{-}t\text{Bu})_{18}$ and $Au_{36}(SPh)_{24}$ structural models.
10. References.

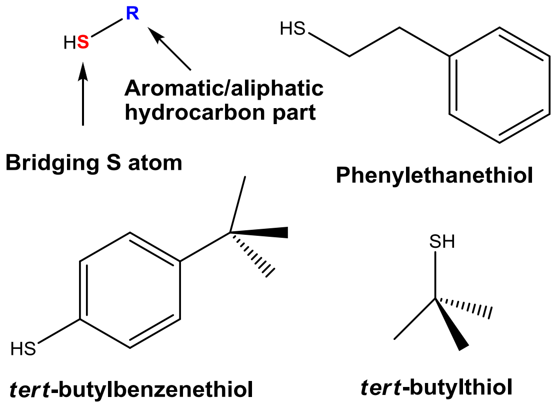

Figure S1. Most widely used physicochemically different three classes of ligands; aliphatic ligands and aliphatic-like ligands (phenylethanethiol) having a primary carbon adjacent to S atom, aromatic ligands (*tert*-butylebenzenethiol) having an aromatic ring adjacent to S atom and bulky ligands (*tert*-butylethiol) having a tertiary carbon adjacent to S atom.

Computational details

Local geometry relaxations and Ab Initio Molecular Dynamics (AIMD) runs were performed using the CP2K code (Hutter et al., 2014) whose DFT algorithms are based on a hybrid Gaussian/Plane-Wave scheme (GPW) (Lippert et al., 1999). Pseudopotentials derived by Goedecker, Teter and Hutter (Goedecker et al., 1996) were chosen to describe the core electrons of all atoms and DZVP basis sets (VandeVondele and Hutter, 2007) to represent the DFT Kohn–Sham orbitals. Calculations were performed spin-restricted and at the Gamma point only. The semi-empirical Grimme-D3 correction (Grimme et al., 2010) was added to Perdew–Burke–Ernzerhof (PBE) (Perdew et al., 1996) exchange and correlation (xc-) functional to take into account dispersion interactions. The cut-off for the auxiliary plane wave representation of the density was 400 Ry. AIMD runs used a time step of 1.0 fs and the temperature was controlled by Nosé–Hoover chain thermostats (Martyna et al., 1992). The geometries of $Au_{38}(SCH_2CH_2Ph)_{24}$, $Au_{36}(SPh)_{24}$, and $Au_{30}(S^tBu)_{18}$ clusters were obtained via fully relaxed local geometry optimizations starting from configurations derived from X-ray measurements in Refs. (Qian et al., 2010), (Nimmala et al., 2014) and (Dass et al., 2016), respectively, after completing the missing atoms of $Au_{38}(SCH_2CH_2Ph)_{24}$ as needed. The Cartesian coordinates of the resulting $Au_{38}(SCH_2CH_2Ph)_{24}$, $Au_{36}(SPh)_{24}$, and $Au_{30}(S^tBu)_{18}$ species are provided in the Supplementary Information (SI). In the system comparison analysis, we use clusters for some of which no crystal structure is available (or have not even been synthesized yet). For these systems we create initial geometries starting from the experimental structures in Refs. (Qian et al., 2010), (Nimmala et al., 2014) and (Dass et al., 2016), we perform local optimizations, and follow these by three sequential AIMD runs lasting : (a) 2 psec at

300 K, (b) 2 psec at 900 K (only in this AIMD run the coordinates of the Au atoms were left frozen to the initial geometry), (c) 2 psec at 300 K, leading to a final full geometry optimization. This long procedure is deemed necessary to equilibrate structure and ligand-ligand interactions especially for $Au_{38}(SPh)_{24}$, and indeed the total energy of this system decreased by 3.7 eV after the equilibration protocol, while a smaller decrease in energy was observed for $Au_{36}(S^tBu)_{24}$ and $Au_{30}(SPh)_{18}$. (1.5 eV and 0.6 eV, respectively).

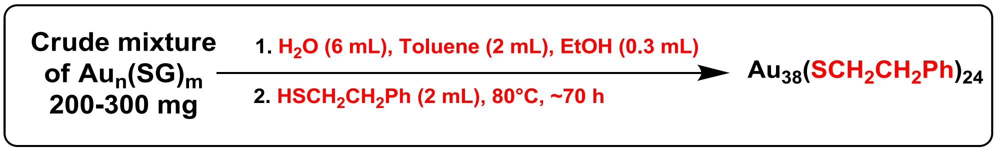

Scheme S1. The protocol for the synthesis of $Au_{38}(SCH_2CH_2Ph)_{24}$.

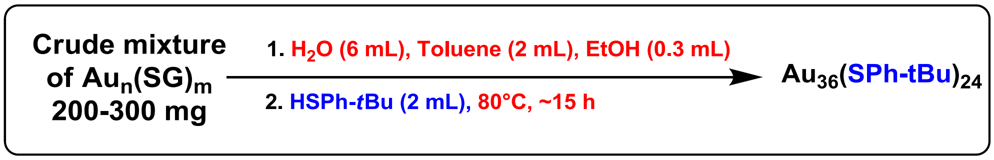

Scheme S2. The protocol for the synthesis of $Au_{36}(SPh\text{-}tBu)_{24}$.

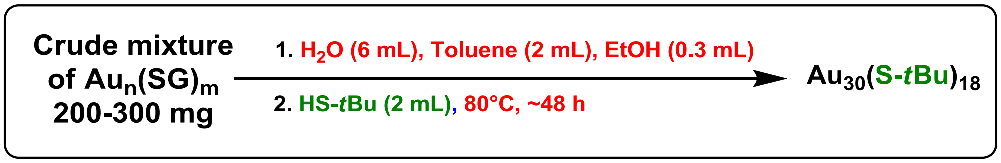

Scheme S3. The protocol for the synthesis of $Au_{30}(S\text{-}t\text{Bu})_{18}$.

System : $Au_N(SR)_M$	N,M=30,18,R=tBu	N,M=36,24,R=Ph	N,M=38,24,R=EtPh
$Au_N(SR)_M$	1687.98201666	2332.90823832	2730.59465752
Au_N	996.60577862	1195.91865934	1262.35730340
$(SR)_M^{crown}$	689.039641560	1134.25963456	1465.20378111
$SR_{relaxed}$	38.27338148	47.25499543	61.00837653
$HSR_{relaxed}$	38.91610700	47.88447318	61.65151849
Au_{atom}	33.1443376704	33.1443376704	33.1443376704
$Au_N(SR)_M$-anion-adiab	1688.04648476	2332.98786144	2730.67891792
$Au_N(SR)_M$-cation-adiab	1687.79592159	2332.70818507	2730.42134317

Table S1. Absolute electronic energy values for the $Au_N(SR)_M$ systems here considered. The nomenclature is the following: $Au_N(SR)_M$ = full nanomolecule; Au_N = gold cluster in the interacting configuration; $(SR)_M^{crown}$ = crown or shell of ligands in the interacting configuration; $SR_{relaxed}$ = fully relaxed SR thiyl radical; $HSR_{relaxed}$ = fully relaxed HSR thiol; Au_{atom} = isolated Au atom; $Au_N(SR)_M$-anion-adiab = total energy of $Au_N(SR)_M$ anion in the geometry of the neutral species; $Au_N(SR)_M$-cation-adiab = total energy of $Au_N(SR)_M$ anion in the geometry of the neutral species. All energies are in atomic units.

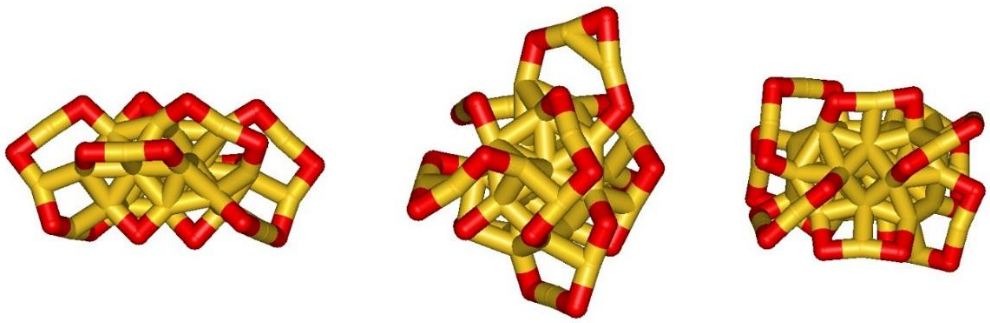

Figure S1. Schematic pictures of the $Au_N(S)_M$ skeletal framework of $Au_{30}(S^tBu)_{18}$, $Au_{36}(SPh)_{24}$ and $Au_{38}(SCH_2CH_2Ph)_{24}$, respectively, from left to right.

Model	Allometric1	$Au_n(SC_2P)_m$	
Equation	y = a*x^b		
Reduced Chi-Sqr	11.48284		
Adj. R-Square	0.97807		
		Value	Standard Error
B	a	3.43606	0.68056
	b	0.55675	0.03821

Model	Allometric1	$Au_n(TBBT)_m$	
Equation	y = a*x^b		
Reduced Chi-Sqr	1.49222		
Adj. R-Square	0.99697		
		Value	Standard Error
B	a	2.85048	0.19808
	b	0.60018	0.0138

Model	Allometric1	$Au_n(S\textit{t}Bu)_m$	
Equation	y = a*x^b		
Reduced Chi-Sqr	0.16585		
Adj. R-Square	0.99525		
		Value	Standard Error
B	a	2.48161	0.23067
	b	0.5896	0.02424

Table S2. Standard values and errors associated with the nano-scaling law.

Cartesian coordinates of the $Au_{38}(SCH_2CH_2Ph)_{24}$ structural model.

Au	19.5142016581	19.7735751436	20.8373717685
Au	19.2877131421	23.2477030939	22.1799950146
Au	18.9079447561	20.2542770805	18.1048550066
Au	19.6473338542	17.4956383716	19.1406857806
Au	18.9488701929	14.8188983150	19.7497403561
Au	18.9161608748	15.8010840510	16.8064870956
Au	18.7531823264	20.7784206018	15.2879143141
Au	18.7623609724	25.6328344120	19.5750357937
Au	18.3132082231	23.1135005704	17.2489474898
Au	18.6811410516	13.7823156938	22.8598602434
Au	20.1608582866	23.1799187812	14.2963344526
Au	20.3228160504	22.3466905700	19.3655670601
Au	20.2206557862	18.3428556876	16.4249219125
Au	18.1327422056	11.5357297387	20.3139213346

Au	17.7451028793	17.1867826788	21.4053523917
Au	17.1898830697	13.2903780376	14.6816684733
Au	17.3904616243	18.5151441988	16.3900210814
Au	17.5549376076	22.1938859258	19.9111259477
Au	17.3509632614	18.2236823178	13.4903855737
Au	21.2858411576	21.0466418654	16.8262955533
Au	21.7401801354	19.4002473221	19.0953759652
Au	16.9352567445	16.7616212621	18.6319356518
Au	17.0393682322	13.8937134577	17.7475451685
Au	16.5029734907	23.3125479547	14.9245323182
Au	16.7635684499	19.3916466848	19.8277716419
Au	22.1148076131	16.1537003271	20.2953100075
Au	16.2641366175	20.9930169621	17.4763298680
Au	16.2276294223	14.8542033180	20.5106240840
Au	16.0382059251	15.9905519456	16.0174261805
Au	14.9125288474	21.0327693771	21.5059609974
Au	23.6028052285	21.8462857046	18.7141905332
Au	14.8019954385	17.4567217176	20.5518576578
Au	14.8138905939	18.3539477468	17.6199978909
Au	23.5927047693	18.7471457704	16.7689129332
Au	14.2426342925	15.5954154561	18.2925120907
Au	14.0571823568	12.9755932362	15.8074113235
Au	12.9757846861	14.8690242237	21.3543563727
Au	11.6566655401	17.5712805252	19.2858917800
S	19.2495478128	25.6019690149	21.8671467652
S	19.5370819871	20.9789724426	22.9146041466
S	19.1723762840	11.4372596711	22.4175164653
S	18.8304931420	19.9961886045	12.9241073484
S	19.3299678884	14.2823823377	14.9089147924
S	18.4313787148	16.0829554851	23.4740070837
S	18.1816914725	24.1976808380	13.5795628418
S	18.2541512729	25.5829328769	17.2984660358
S	21.1387205277	14.0020703136	20.3786614977
S	17.0151503160	11.5241379462	18.2585733879
S	22.3006903421	23.7349694768	19.2936246615
S	22.2168902068	22.2394976692	14.9739806797
S	22.2066135438	17.2666735556	15.5734002462
S	15.7892259896	16.4493753805	13.6585403369
S	15.7172460243	23.2674815939	21.1418437682
S	15.3970466554	11.6714373584	14.3007079278
S	23.5149968360	18.0499300738	20.1983333410
S	14.7295169731	22.2563190441	16.0642161683
S	14.8497088129	13.5847677824	21.9500892910
S	13.8254344321	19.0249244203	22.1378392365
S	25.0969422528	20.2500644531	17.7924011329
S	12.6851140923	13.9241634269	17.4718439739
S	12.5014973784	19.0907851921	17.6878177665
S	11.0295715817	16.1142274754	21.0281163568
C	19.8117967379	13.5789155562	27.1069124983
C	18.9104286851	23.4550189556	9.5836149592
C	20.4101372088	8.1442631240	19.7904034696
C	18.9130614391	24.1586332641	10.9122376411
C	18.9824917731	22.0638512423	7.1413429939
C	20.0917600432	8.1499260459	21.1538582837
C	18.1297115130	21.6332495197	25.8879717639
C	18.8859145702	23.4612419296	7.1477178452
C	19.0717502867	17.7338725659	9.7368627397

C	20.7633164797	16.2081020622	27.0632299967
C	19.9023327072	25.9219067221	16.5878271706
C	17.6071170041	21.1224780643	27.0884509560
C	18.7466006168	16.7048582757	10.6331365924
C	18.8455994660	24.1490913562	8.3681943171
C	19.0137361939	22.0532078046	9.5687547379
C	19.0440301728	21.3652273498	8.3560728207
C	18.3285761153	21.2122411006	28.2858649040
C	19.4111201932	22.2147730208	25.9085598679
C	19.3964854988	14.7640297085	12.1052485718
C	19.7279623409	15.7973473250	11.0516357098
C	20.6980818779	15.4764506033	25.8612586640
C	20.0350134063	27.4966129718	16.5929018626
C	19.5931179436	15.4128543607	13.4867619505
C	20.3970084899	17.9062569917	9.3326783092
C	20.2290707184	14.1553420279	25.9050579392
C	18.4279232014	8.4333136870	23.0215216485
C	29.0390342255	23.7806857497	14.5998564412
C	18.7499603630	8.1680108093	21.5800390734
C	20.5349258543	19.2741235682	12.7287440608
C	19.3896877119	8.1473360749	18.8313010569
C	9.3796854286	20.0222591671	21.7019324591
C	20.0712703460	16.8926477637	23.7904405693
C	8.0874072952	12.7453666129	16.6972164039
C	8.1042027260	10.3183066466	17.0464997429
C	8.0896864292	13.0950369715	21.6335829472
C	17.8453667594	23.6099016149	11.8751194884
C	19.8894903638	14.3105769200	28.2972069324
C	7.4341942507	11.5391205875	16.9821176961
C	7.5430155240	12.6497489559	20.4152789273
C	7.5150881963	12.6160863239	22.8272802139
C	6.3920947212	11.8033832686	20.3865708993
C	6.3738343506	11.7705758202	22.8185044585
C	20.3722954723	15.6264745761	28.2764047787
C	5.8605973895	11.3724336405	21.5914691959
C	17.3394606519	21.5632914849	24.6041246468
C	17.8713935429	20.5580408231	23.5774856619
C	18.6556162994	6.6106224389	15.6505011417
C	18.1559508990	10.0752422357	23.2992269511
C	18.1844250720	7.9513684269	15.5875265921
C	17.6262343224	26.1824811908	22.5392113752
C	19.6046302361	21.7903792111	28.2913652574
C	21.4147084115	10.4874154792	16.5529996341
C	20.1483073861	22.2780748652	27.0963008368
C	17.9084696098	27.7676303307	22.5460721109
C	21.1237631471	16.0515307059	24.5306689798
C	19.0929779032	5.9575607247	14.4935114874
C	21.0561111901	15.9861601559	10.6455608667
C	21.9117054431	10.1369006855	15.2923775614
C	21.3192593416	27.9302613283	15.9556876487
C	21.4634416417	20.2240598564	11.9449330473
C	21.6825164551	11.6986127827	18.7892518191
C	18.0505601506	8.1394604229	19.2447502330
C	21.3838616629	17.0075245817	9.7425056005
C	21.3068536788	28.8567337846	14.8787212955
C	18.0573713014	8.5324147182	14.3330607045
C	17.7325111320	8.1444553720	20.6090879836
C	17.5204231497	9.9719718036	14.2532888571
C	22.1858455884	11.2810894610	17.4302385226

C	19.0637064437	6.6217205333	13.2628507202
C	21.7159089283	13.2513383309	18.7895535456
C	18.5890062566	7.9596033030	13.1822681106
C	21.8078685435	25.8402772477	11.9656537344
C	16.7448034638	28.8219181152	22.4563022280
C	22.5611924817	27.5687777994	16.4956239152
C	15.9186295559	28.9998132837	23.5856662958
C	22.3284914625	25.4117525607	10.7462062892
C	23.2075128020	10.4951085489	14.9056264158
C	22.5085472727	29.1632264755	14.2389579958
C	16.1696804248	29.0002020117	21.1777071282
C	22.4145682145	25.4884649032	13.1689508476
C	22.9245923413	19.7914604990	11.9468969273
C	16.1939010709	10.1873375872	15.0050516567
C	22.7340036874	24.2342695432	21.0157162575
C	22.4429063485	15.7040745990	16.5067479239
C	23.4966185455	11.5959325080	17.0402901111
C	23.6441952088	19.6800742397	10.7472276032
C	23.3106634166	24.3999625751	10.7544479968
C	23.9645601632	11.3160009778	15.7579133376
C	23.1749899166	23.6874876233	15.5918864310
C	23.7434039721	28.8468569397	14.8082749450
C	14.9646714272	20.2812186775	11.8538716887
C	23.7715454983	18.6575042883	21.9278599580
C	15.2479257038	14.1701071504	23.6497338468
C	23.6190802972	19.5215090260	13.1390870970
C	14.5259670198	29.0555573434	23.4416245891
C	23.4434333000	24.5215234612	13.1820320751
C	15.0418502056	19.2645074364	10.9019830717
C	23.8529918177	23.9585989570	11.9651666667
C	15.2611758640	11.4867027516	18.8045141615
C	23.3537931213	20.1229099380	22.1251296984
C	23.7634011901	27.9276600234	15.8867019842
C	14.6464454874	18.5338108189	23.7215146135
C	23.5405949586	25.5767214458	21.0950748832
C	14.6088675939	25.4624994687	19.6528036711
C	14.8758276434	10.2767828652	19.6526240401
C	23.2106992893	20.4241939890	24.6547083761
C	14.7781317234	29.0538748501	21.0293927974
C	14.2597479509	21.0138762441	14.7886888168
C	14.7090413431	11.5227791846	25.7787234122
C	23.7059241340	14.9692035442	16.0459419029
C	14.6417584859	23.9046104844	19.7783301002
C	13.7120331851	21.6789645539	13.5130377037
C	24.1118012020	24.1813080536	14.4931072196
C	14.1397228561	17.2547743385	13.6601708481
C	13.9495970673	29.0318944064	22.1618933986
C	14.7257603366	10.1284285985	25.7802292548
C	13.7266524615	20.6869085575	12.3776274723
C	14.1831208410	13.7381407595	24.6729963818
C	24.9983355259	19.3151791569	10.7348758981
C	23.8666471568	20.6594764710	23.4377582231
C	23.7729514106	20.8451780416	25.8663166232
C	14.0098692255	25.6566661210	17.2026986881
C	24.9654403009	19.1405543240	13.1340732176
C	14.0680273095	12.2331851488	24.7496827895
C	13.8691723229	18.6405071836	10.4646108995

C	13.6020814734	25.6685320728	18.5463510250
C	13.7868714188	17.4846210725	24.4442262417
C	25.6630134236	19.0332794369	11.9281562880
C	24.9793080672	25.3737823950	20.6536828667
C	13.3901313245	10.2812135290	19.9467921090
C	12.7143153605	9.0832102433	20.2119323763
C	25.0185410571	15.6915489377	16.2433411133
C	14.1188353843	9.4138338868	24.7372593365
C	13.0478046948	16.2657185158	14.0969837497
C	13.0603739265	25.6477618784	16.1742198421
C	25.6077951601	15.8067881109	17.5126823325
C	25.5274704738	25.8087441381	19.4429895187
C	12.5582522648	20.0864013554	11.8933186236
C	12.6254442405	19.0806258279	10.9223807779
C	12.7114847597	20.7432927508	18.4700217958
C	13.3984677352	11.5088874286	23.7515299018
C	25.5873957708	21.2408534954	16.3146369937
C	25.0962199469	21.3366614185	23.4627547007
C	25.0106985592	21.5042378671	25.8768815568
C	12.2238047459	25.6575036876	18.8334600924
C	25.6947367855	24.3827531238	21.3701059638
C	13.4427167644	10.1176419326	23.7351031255
C	12.4206854056	17.9919864436	24.8509338388
C	25.6641321254	16.2565841644	15.1355860556
C	12.6564216280	11.4796016413	19.9359085404
C	12.3072948445	19.1129680562	25.6899143801
C	11.3420421783	9.0800935482	20.4711894448
C	25.6596620929	21.7656271603	24.6656025024
C	11.6885583283	25.6442758447	16.4689888133
C	26.8317245382	25.4523762060	19.0459432350
C	26.9895973879	20.8436734981	15.8285929870
C	11.6922504940	16.7942667732	13.6877205058
C	26.8660563511	16.4216022809	17.6627555418
C	26.9694347911	22.1935387934	13.6501921721
C	11.2684359054	25.6484821130	17.8065880341
C	11.1600946558	14.7850998071	16.8747194712
C	26.9336753003	23.9121919418	20.9258671669
C	11.3941969562	21.4534427749	18.8059447881
C	11.0844610329	17.8221505104	14.4221543155
C	11.2438345417	17.3408498325	24.4596235370
C	11.0551120440	19.5701347458	26.0964998686
C	26.9175453675	16.8754238605	15.2776288475
C	11.4789408459	21.7994149078	21.3095037800
C	10.6177171130	10.2735175031	20.4311267013
C	27.4848679010	16.9829194006	16.5466728292
C	11.2787701239	11.4737886122	20.1479820320
C	27.4744756246	24.4185303017	19.7414260541
C	27.4319686920	23.2664193739	12.8789153637
C	27.4999302564	21.9298436387	14.9213474216
C	11.0894347689	16.3545246866	12.4995679372
C	10.1790459631	14.0004878364	15.9602832564
C	10.9134534856	21.1586185716	20.1986425694
C	11.0087351538	21.5603431081	22.6011251942
C	10.1409319603	11.5151335603	16.5377503660
C	9.8850199233	18.3972202025	13.9814100449
C	9.8796859186	18.9053810328	25.7071697681
C	9.9785704634	17.7980179431	24.8564728171
C	9.6117338967	14.9691620716	20.6076014452
C	28.5761894266	22.7195364540	15.3732625912

C	28.5295774931	24.0256545262	13.3256095012
C	9.4762095162	12.7517259915	16.4553961556
C	9.8950765525	16.9321258440	12.0442488069
C	9.3809396365	13.9481697914	21.7363663332
C	9.3239878264	17.9776224996	12.7735629011
C	9.4897244247	10.3147833311	16.8394640567
C	9.8501476217	20.2639073290	20.4103821090
C	9.9130683958	20.7062856642	22.7925953451
H	19.2314419904	8.1255834820	23.7003871481
H	19.1564523356	27.8630657614	16.0534919551
H	19.4496470286	12.5531621945	27.1146812989
H	18.3542121075	14.4255984077	12.0106925726
H	19.0084277041	21.5340909133	6.1866073401
H	18.9274807828	16.2836864542	13.5944914210
H	19.9271815224	25.5661631548	15.5501264422
H	18.7970836558	25.2539725291	10.7992847482
H	17.9139284231	20.8686733954	29.2439699987
H	21.1411509745	17.2304964715	27.0553686321
H	18.8383427233	23.9973812067	6.1970520424
H	19.8239306391	22.6276317978	24.9882873859
H	20.3959613124	10.2237732496	16.8335733435
H	21.4543465906	8.1429637774	19.4706246396
H	19.8013167206	17.7774078505	24.3871074013
H	19.9640302568	27.7985258346	17.6481265110
H	19.0672207558	21.5120560118	10.5181303208
H	20.6399698279	18.6593385445	8.5735066688
H	19.1173518319	20.2794495099	8.3555913383
H	19.8993909688	24.0103379640	11.3836924891
H	20.4197854109	18.3210672345	12.2014271464
H	20.8913117943	8.1578049824	21.8985928250
H	18.5437473316	27.8832191253	23.4353202667
H	19.6469545989	8.1457440596	17.7710449683
H	9.1044892635	17.2554041164	24.5203281915
H	18.7710204470	25.2435938729	8.3777172126
H	20.6483496303	11.3865300678	18.9857277778
H	9.4262871899	19.7460202179	19.5517438046
H	9.5242608321	20.5378739052	23.7914720178
H	8.3673319940	18.4024309826	12.4471748488
H	7.5348789782	13.6804853953	16.6355968577
H	29.9052485474	24.3459007248	14.9521231579
H	16.6151352154	20.6714340521	27.0872491518
H	7.9818429967	12.9680795767	19.4699053227
H	18.2977966437	18.4435905369	9.4538824373
H	7.9404429257	12.9328592850	23.7788511635
H	18.6726613769	6.0805104485	16.6034977149
H	8.5125523015	19.3710495216	21.8451366576
H	7.5732808968	9.3937955729	17.2535708298
H	20.7072622885	25.4957212902	17.1882537785
H	6.3407805923	11.5469633317	17.1141373322
H	17.3093025591	22.5559784856	24.1258762511
H	18.5605390149	27.8832743801	21.6683492266
H	20.3425961896	29.0922115785	14.4294892012
H	17.8279779942	22.5143183610	11.8798400305
H	17.7104921023	16.5693515583	10.9393498444
H	17.7379939269	8.3235099676	16.5017872650
H	21.2889024291	9.5481364541	14.6222631495
H	6.0242578948	11.4829466012	19.4167249266

H	5.9759028652	11.4454122891	23.7738994262
H	17.4539706553	8.0489677845	23.3469262405
H	20.0398520475	13.8790825918	12.0041082728
H	4.9633693431	10.7408129364	21.5755747683
H	20.9171024623	19.0704788140	13.7342408983
H	18.2758296700	10.6518773499	14.6698664127
H	17.9784559903	19.5540839978	24.0108243608
H	17.5082130620	25.7462863045	23.5421752813
H	18.3022464229	10.2627099363	24.3718843514
H	19.4118710385	4.9110163379	14.5441828157
H	21.1152257011	20.3027939620	10.9041300468
H	20.6218579395	15.7822035489	13.6026430763
H	20.1992622484	13.5789361169	24.9774700491
H	19.6114881159	13.8763697858	29.2662500339
H	16.8384846715	23.9717131417	11.6310961824
H	20.4726648591	17.2321323212	22.8263934421
H	21.3934566660	21.2358956438	12.3788908861
H	17.1771493725	20.4869657462	22.7279517324
H	20.9865161537	26.5563799374	11.9767851116
H	20.4535554919	16.1617819293	29.2324059102
H	21.4326601193	15.2342350464	23.8608692901
H	16.2909392231	21.2916560515	24.8020054402
H	21.1385089996	22.7305521296	27.0919744456
H	22.3257843788	11.3647959653	19.6135118827
H	20.1280268450	21.8768823717	29.2541216357
H	21.0941256870	13.6418265249	17.9769345145
H	16.8163084924	25.7995957957	21.9048841532
H	17.1055802723	10.2766803955	23.0632874448
H	16.3597021784	28.9631724183	24.5843690875
H	17.3947416644	10.2543777045	13.1998220633
H	22.0046698663	16.7019687628	24.6602790388
H	21.8288685613	15.2888275839	10.9625420133
H	21.8941528664	25.7062893541	9.7876714055
H	21.7822532188	24.3098316486	21.5573564023
H	19.3938161498	6.1030939603	12.3631171920
H	21.5584767555	15.0884103673	16.2975980755
H	22.4236316543	17.1494847489	9.4610061836
H	17.2442869164	8.1380191071	18.5070610258
H	16.8085998573	28.9636030614	20.2915633673
H	16.6868685881	8.1490730059	20.9282572133
H	22.5812052265	26.8949423790	17.3542958486
H	22.0218674159	25.8248944353	14.1252483390
H	18.4704611374	8.3471782065	12.1765830106
H	15.8798548486	20.7474925955	12.2199227756
H	22.4827051718	29.7910897307	13.3487518433
H	22.4610762219	24.4441873339	15.9232254445
H	16.3462740387	10.2954823797	16.0847158416
H	16.2225260217	13.7113683168	23.8793716019
H	23.5927197082	10.2250736122	13.9245731970
H	23.2573305894	17.9946511940	22.6315201141
H	22.4492660108	15.9272169948	17.5821179059
H	23.1528995213	19.8787793338	9.7895128729
H	16.0249768281	18.9197903851	10.5901673614
H	22.7370173495	13.6128657310	18.6332620029
H	22.2430574875	19.9215995681	24.6469766453
H	15.6274361884	25.7849803632	19.4012836509
H	15.6520944145	18.1501266329	23.5051086078
H	23.1107270729	19.6159913497	14.0964265439
H	22.2573845513	20.1903008071	22.0608045381

H	23.3073615271	23.4259818243	21.4775046187
H	15.1462329594	20.4089140085	14.5630106958
H	15.3866451831	15.2559623837	23.6287845460
H	15.1983666824	12.0479989610	26.5983571387
H	15.4554109000	10.2950719212	20.5921985620
H	14.3623879172	22.5328693542	13.2644802376
H	23.0291954250	26.3813247401	20.5543780580
H	24.1274185652	12.1393404451	17.7405541593
H	23.6600997520	23.9947133859	9.8016660814
H	15.1457378029	9.3445174199	19.1390687193
H	15.4987127915	9.3583012523	14.8240149210
H	14.7460016868	19.4407764535	24.3320059096
H	24.8552032127	18.5463969668	22.0933205081
H	23.7313249438	23.3662427064	16.4824794104
H	13.8950464807	29.0556633048	24.3334903993
H	15.2104737738	9.6089413150	26.6046307094
H	13.9682418084	17.6022924146	12.6381139682
H	15.0758452739	25.6408622743	16.9616829582
H	14.2955098907	25.8507379034	20.6261800837
H	24.6822528360	29.0887626949	14.3118232788
H	15.0777115309	12.4210476759	19.3432262777
H	23.7275175693	14.0123485150	16.5861620941
H	23.5288223398	25.7978227513	22.1749298634
H	24.6285236389	23.1943212396	11.9622016391
H	24.9859144599	11.5765936125	15.4799001412
H	23.5963238842	14.7068999612	14.9839570911
H	23.2694701701	20.6516639452	26.8161982631
H	14.6663185943	11.5126903460	17.8754401032
H	14.4754873440	14.1413898141	25.6572052504
H	14.9759713690	23.4570324455	18.8352565591
H	23.7490068631	20.7250068101	21.2909566810
H	14.3412298205	17.1447501419	25.3354950343
H	13.2788598210	8.1523471211	20.2119744909
H	14.3415623066	29.0516403856	20.0285606691
H	24.7218953729	27.6497980246	16.3240056023
H	24.6028488769	25.1051452884	14.8636039185
H	13.2280142053	15.2797171588	13.6444757702
H	13.5147530340	20.3525547040	15.2525627798
H	13.9235691998	17.8522367367	9.7113841166
H	24.9161577034	23.4540191261	14.3295561284
H	25.0961548144	15.3947667642	18.3835832511
H	25.5078168953	19.2411075803	9.7707568551
H	12.7047762649	22.0785155729	13.6894129226
H	14.1635707169	18.1256044111	14.3239899873
H	13.6275556458	23.5396091421	19.9732653281
H	24.9699049562	26.5274910810	18.8406071008
H	13.6697663755	16.6102335364	23.7848642684
H	12.8650845665	29.0112996442	22.0455420518
H	13.3990523010	25.6345208858	15.1379502270
H	25.4475720287	18.9148094843	14.0829651130
H	24.8370452331	21.1375174827	15.5257887115
H	13.2169437579	14.1979898046	24.4142343841
H	13.3594296716	20.6481345335	19.3532915970
H	13.2721580517	21.3092516626	17.7183179013
H	13.2059604720	19.6469324558	25.9927569427
H	14.1434637014	8.3136775252	24.7206886825
H	26.7249787839	18.7648958363	11.9180786711

H	13.1261351878	16.1256193234	15.1854638667
H	25.2710591402	23.9828674296	22.2909187463
H	25.1947881659	16.1916531932	14.1548314410
H	13.1350465806	12.4304571523	19.7263776094
H	26.1411708077	21.5846763511	13.2822613227
H	26.9818851602	19.8574495232	15.3423087889
H	11.8943094271	25.6502434400	19.8741888675
H	11.5881505013	20.3779133904	12.2956348932
H	25.6023144941	21.5233466297	22.5166490575
H	25.6025149802	22.2879292415	16.6363769435
H	12.8375697639	12.0026013918	22.9631864428
H	25.4446324130	21.8099108560	26.8310702090
H	12.3102071674	22.4894319234	21.1633213135
H	11.7017388220	18.5943795192	10.6181044609
H	11.4517362788	15.6990035594	16.3465324140
H	10.8365404617	8.1452573372	20.6924770802
H	12.9340461318	9.5927043122	22.9306779005
H	11.5351330868	18.1729194631	15.3499805992
H	27.2386215548	25.7808489296	18.0929209766
H	11.5546315385	22.5379914603	18.6941940225
H	27.3002848070	16.5062238176	18.6557930850
H	27.0039782871	23.4676271374	11.8984985383
H	10.9699806908	25.6412994572	15.6529423209
H	10.9985580950	20.4181724349	26.7800791258
H	10.7651308251	13.7373478913	15.0622975400
H	11.3152062302	16.4796844115	23.7964761048
H	11.5534790107	15.5543382935	11.9252762084
H	27.6175204188	20.7506754665	16.7193891282
H	11.2168203344	11.4988912814	16.3469267976
H	26.6351684575	22.2598207591	24.6587661057
H	27.4246277597	23.1189944533	21.4826535283
H	10.6344375659	21.1806095764	18.0632834172
H	10.6966236141	15.1039967361	17.8174589456
H	11.4559446221	22.0687900517	23.4522356063
H	10.1994013962	25.6447864763	18.0445616708
H	27.3962012584	17.3066283797	14.4016071043
H	9.7929017784	14.4777573488	19.6439975969
H	10.2360204921	13.2639398590	21.7906768518
H	10.7352672597	12.4077441220	20.0571606092
H	9.5427089597	10.2730718729	20.6031126905
H	28.4781317895	17.4361560896	16.6540322003
H	28.4619401632	24.0752595282	19.4109870212
H	9.4418839428	14.7456424775	15.6284043751
H	9.4381623618	19.2004342093	14.5631285440
H	28.9507614047	22.5379726676	16.3779834869
H	10.0428878834	9.3823340917	16.8898928285
H	28.8895532198	24.8567047247	12.7240238961
H	9.3826081511	14.4813446497	22.6995776965
H	9.4594227292	16.5976891318	11.1024006055
H	8.7408431520	15.6296072580	20.4942693048
H	8.9119256493	19.2733099461	26.0302195001

Cartesian coordinates of the $Au_{30}(StBu)_{24}$ structural model.

Au	24.5871587821	15.7186498387	15.7992224398
Au	24.2208849353	19.1345538864	15.7953924506
Au	22.8298923199	13.9011520874	18.3299283458
Au	22.0561284833	17.0166443029	14.9767024585
Au	22.1747945842	17.2113645390	17.7236655309
Au	20.7356745562	15.1743022498	16.5889857758
Au	20.2083256099	15.8780634511	19.4963527823
Au	20.5882298677	19.5740311837	16.4065176550
Au	20.5815288036	19.8698282089	19.5308866939
Au	10.7219476359	19.7537520622	17.9740751594
Au	19.5793114102	17.5240406212	12.3908765735
Au	19.2682400537	17.3703775834	15.2366613760
Au	18.8943144053	17.7775281470	17.9618506294
Au	18.3097905294	17.5771357480	20.6620958779
Au	17.5221113025	15.3307796084	19.0817429737
Au	17.5784413530	15.0590534906	15.8340962974
Au	17.7843210813	19.6610027365	16.1261634811
Au	16.9545604135	19.9779909627	18.9453155013
Au	16.6055212425	17.2174749552	14.1636422668
Au	16.1328632921	17.3332569247	16.8393571875
Au	15.6414915684	17.5388601583	19.5387862226
Au	15.1626938340	18.3821805936	22.1797841329
Au	14.4601950886	19.4133131086	17.8401326813
Au	14.8791635982	15.2980899541	15.3226482049
Au	14.5778642376	19.5469758359	14.7505248149
Au	14.1931556726	15.3677862486	18.3165434709
Au	12.7737866466	17.4455669263	19.5966727373
Au	12.7579797000	17.2711352855	16.8564372203
Au	12.0333966536	13.9759151271	16.7963919175
Au	10.2277518074	16.4823336485	18.4441319843
S	25.9697375950	17.6141434083	15.2983325598
S	23.8436964504	13.5119070075	16.2367515808
S	22.6591561242	20.8139195670	16.3483062682
S	21.8399164359	14.3377133568	20.4223285944
S	21.9134497894	17.7175148622	12.6596497730
S	22.7350435688	18.9078210949	19.3345039550
S	18.6979489272	21.1751557298	20.0791700608
S	17.2734476849	17.4766372678	11.8782913621
S	19.1063542374	13.3919852775	16.5623897949
S	16.1500813584	21.2384260577	15.2490095990
S	17.4700016236	18.5308763555	22.6903385982
S	15.8789013081	13.6635109542	18.6053028258
S	13.0625519933	17.7709880764	14.4862886697
S	12.8223161547	18.3605355506	21.8527970415
S	13.0744233898	13.8483811212	14.6835166805
S	10.8988986217	14.2361431192	18.8605804490
S	12.6749498455	21.0367701782	17.5666701261
S	8.8250922865	18.3310088180	17.8597964789
C	27.2401550066	17.7316196442	16.6921217083
C	26.5385425143	17.7288197949	18.0548409640
C	28.1709146935	16.5160111020	16.5103299276
C	28.0257683553	19.0377196392	16.4371807947
C	25.3737314795	12.4914386529	16.5581995357
C	24.8994075476	11.0964585418	16.9911400954
C	26.2383080219	13.1384243673	17.6423926095

C	26.1150742942	12.4288657359	15.2136142353
C	22.6618190532	22.0097783269	14.8995049526
C	21.7822457131	23.1914979195	15.3358976722
C	22.1015382083	21.3488197568	13.6385537502
C	24.1202869108	22.4713610491	14.7107140329
C	20.9146447374	12.7961496493	20.9556326211
C	20.1773358373	12.1536540974	19.7838722889
C	21.9735166202	11.8393428875	21.5248945919
C	19.9357386530	13.2459172647	22.0500398943
C	22.6304651656	16.3429019437	11.6014721745
C	24.1512190451	16.4416572320	11.7890740086
C	22.2343665931	16.6359108835	10.1465059515
C	22.1183531080	14.9709533586	12.0386514325
C	23.2563474403	18.2990693002	21.0380656385
C	24.1625104753	17.0767111901	20.8454862657
C	24.0657520090	19.4627485129	21.6474672674
C	22.0499365931	17.9705159622	21.9172570078
C	18.9508955795	22.8716129628	19.3022412165
C	18.7114373585	22.8402301207	17.7943299848
C	20.3868981090	23.3160810442	19.6179184698
C	17.9320403933	23.7846774073	20.0040522851
C	16.9086102984	15.8075843656	11.0927156549
C	17.4357348118	15.8934461074	9.6526309703
C	17.5764212807	14.6558044436	11.8446662198
C	15.3793268059	15.6559850071	11.1075816990
C	19.5504916627	12.1780631046	15.2083298088
C	18.2481202158	11.4961386554	14.7709118751
C	20.2335143240	12.8794789111	14.0373348037
C	20.5028646823	11.1703179327	15.8747121949
C	16.8650551472	21.9493399093	13.6663471075
C	17.3646288497	20.8600621505	12.7203073452
C	18.0286402900	22.8469613755	14.1129980443
C	15.7417619954	22.7706410464	13.0152731862
C	17.7763324399	17.2304932163	24.0138177068
C	17.2199761134	15.8653680919	23.6057501211
C	17.0984667998	17.7470461437	25.2906799690
C	19.3000192033	17.1544114644	24.1888585835
C	15.4409562445	12.6915305454	20.1459183805
C	16.6308660844	11.7721825055	20.4480994190
C	15.1675449265	13.6273485601	21.3180594539
C	14.1917863889	11.8716517271	19.7849412757
C	11.4575873347	18.2336630233	13.6460316593
C	10.6201349236	16.9490338160	13.7445665478
C	11.7923300924	18.5686354303	12.1849304148
C	10.7484579532	19.4068049508	14.3238473519
C	12.1775385108	17.0609928590	23.0452669849
C	12.8309852414	15.7018426594	22.8066691795
C	10.6687216338	16.9645267053	22.7830736099
C	12.4642461526	17.5691829222	24.4662312559
C	13.8111527248	12.1337322640	14.4783681712
C	14.6180211396	11.7201255329	15.7082706848
C	14.7057104190	12.2123440135	13.2317268678
C	12.6342910474	11.1743932522	14.2461807403
C	9.3421641788	13.2086178199	18.7484316366
C	8.5252105003	13.5860154846	19.9931170004
C	9.8008669407	11.7430840570	18.7819631751
C	8.5607501665	13.5310937737	17.4721604129
C	12.9011332913	22.2725430573	18.9551675304
C	11.6887400430	23.2148052552	18.9024367878

C	12.9984607484	21.5730547312	20.3111854856
C	14.2002821148	23.0286471495	18.6329229943
C	7.5709376560	18.6416865073	19.2343032911
C	6.7476472034	19.8562550417	18.7522610839
C	6.6904911428	17.3747920796	19.2799373323
C	8.2848902795	18.9033382573	20.5651906316
H	25.8757910511	18.5950411103	18.1618030888
H	25.9584438771	16.8081498265	18.1894032137
H	27.3000713634	17.7759007325	18.8500499164
H	27.8571105239	15.6401136335	17.0894360164
H	28.2240035023	16.2147823561	15.4558972325
H	29.1921276020	16.7657069562	16.8393285863
H	27.4309278433	19.9139560404	16.7285865941
H	28.9626392633	19.0438748920	17.0117437192
H	28.2581993125	19.1420673133	15.3699549540
H	24.3059893219	11.1541461240	17.9158942556
H	24.2788023860	10.6232969443	16.2177115410
H	25.7833123123	10.4627997386	17.1735935347
H	25.8051208642	13.0281502316	18.6438823505
H	27.2403596084	12.6835844233	17.6542215708
H	26.3426570992	14.2137085988	17.4459493614
H	26.3408323908	13.4419410530	14.8511610336
H	27.0565238058	11.8719164637	15.3222106241
H	25.5061910307	11.9264287230	14.4509089072
H	22.1715909384	23.6683357170	16.2448777860
H	21.7505592009	23.9457643198	14.5360982584
H	20.7546192397	22.8643974582	15.5363614784
H	22.7123031304	20.4901611613	13.3315444282
H	21.0782443526	20.9852795996	13.8117816845
H	22.0764424859	22.0755493040	12.8132069795
H	24.4959596824	22.9353305166	15.6314120114
H	24.7750002719	21.6283802652	14.4498098171
H	24.1724154594	23.2172070905	13.9036945917
H	20.8789007693	11.8715720795	18.9872547651
H	19.6487317159	11.2510571743	20.1226843987
H	19.4446589400	12.8438354876	19.3463176790
H	22.6938935468	11.5481313026	20.7480854892
H	22.5279408394	12.2978389223	22.3537994208
H	21.4839057771	10.9283458775	21.8998785357
H	20.4556980717	13.7499320943	22.8752734695
H	19.1926517758	13.9431429329	21.6369326033
H	19.4069030500	12.3729019470	22.4594298334
H	24.5155422096	17.4408215277	11.5210982213
H	24.4180088665	16.2386043378	12.8362451839
H	24.6491889050	15.6997832418	11.1489833099
H	22.5520474495	17.6398381514	9.8367624272
H	22.7099880187	15.9015907228	9.4799091495
H	21.1450449923	16.5675905614	10.0166717106
H	22.3957390338	14.7645669622	13.0808356226
H	21.0249774531	14.9154819010	11.9609202702
H	22.5528501627	14.1883157414	11.3994925745
H	23.6743204001	16.2970391607	20.2485339762
H	25.0939442816	17.3521377925	20.3405020524
H	24.4003290938	16.6458844185	21.8280104518
H	23.4520630932	20.3731204876	21.7009920915
H	24.3606646276	19.1965365482	22.6727132595
H	24.9657596044	19.6816865751	21.0596360819

H	21.4274963388	18.8589604097	22.0898688969
H	21.4146935378	17.2034771034	21.4520895367
H	22.3924045425	17.5887628282	22.8902781379
H	17.6733947407	22.5731909523	17.5572681962
H	18.9251285605	23.8281599995	17.3599773329
H	19.3619126202	22.0977397540	17.3102121213
H	20.5950094545	23.2677882080	20.6946457889
H	21.1174845863	22.6801835627	19.0978059487
H	20.5301364217	24.3550424845	19.2864650568
H	18.1012951060	23.8131604072	21.0881951675
H	18.0243659518	24.8089805023	19.6138626276
H	16.9040623326	23.4432083002	19.8239254498
H	16.9816469505	16.7313481318	9.1075951428
H	18.5261821865	16.0269561735	9.6415734580
H	17.1970249256	14.9639219367	9.1153052669
H	17.2592283629	14.6312715964	12.8971558553
H	17.3051682994	13.6993673952	11.3743741076
H	18.6695997198	14.7547509184	11.8222937025
H	14.8867243060	16.4980224136	10.6035619081
H	15.0999862951	14.7311871489	10.5816870239
H	14.9961760652	15.5985369841	12.1368356950
H	17.7221118582	11.0481279528	15.6240681543
H	17.5697065146	12.2193810633	14.3003315685
H	18.4674792277	10.7002625883	14.0440189730
H	21.1882829510	13.3179232084	14.3558141242
H	20.4325171718	12.1618816325	13.2278546647
H	19.6025351116	13.6859413443	13.6447483466
H	20.0254821692	10.6739006902	16.7297041657
H	20.7912201264	10.3979952735	15.1463453028
H	21.4167240064	11.6690340208	16.2270619761
H	18.1507890050	20.2552200678	13.1949306892
H	16.5500098148	20.1860374723	12.4273262738
H	17.7821943668	21.3161782118	11.8108791901
H	17.7010309073	23.6182294849	14.8221257324
H	18.8078320279	22.2424665513	14.5977024047
H	18.4741120137	23.3452029539	13.2398049104
H	15.3661961147	23.5481537001	13.6931029734
H	16.1152876485	23.2585972415	12.1027516597
H	14.8986395398	22.1223068204	12.7393054116
H	16.1320510143	15.9071111875	23.4645914030
H	17.6711776067	15.5308967110	22.6616496198
H	17.4433239061	15.1207227648	24.3837074432
H	17.4762011356	18.7392914018	25.5699985903
H	16.0106019755	17.8218281562	25.1536457315
H	17.2944851919	17.0574131782	26.1244267475
H	19.7303176534	18.1357381172	24.4270300947
H	19.5425251984	16.4638107055	25.0094652527
H	19.7771663261	16.7823408713	23.2718946061
H	16.8941619445	11.1462851096	19.5855679611
H	17.5124597434	12.3618600247	20.7239213911
H	16.3813412490	11.1109025161	21.2912323340
H	14.3470473091	14.3110177633	21.0748107421
H	14.8962420864	13.0504766330	22.2142361172
H	16.0533220315	14.2342177857	21.5449642178
H	14.3815740504	11.2100006749	18.9292769144
H	13.9035327546	11.2442857156	20.6412696916
H	13.3462599317	12.5268087933	19.5329709086
H	10.4719299110	16.6684990815	14.7978285109
H	9.6390539068	17.1111477263	13.2790224547

H	11.1055827346	16.1045532270	13.2389346349
H	12.4099574306	19.4752338660	12.1225999753
H	12.3370107919	17.7488216993	11.6978811765
H	10.8554772330	18.7447236810	11.6318091511
H	10.5539455366	19.1893245576	15.3836556639
H	11.3644266547	20.3153888361	14.2803313899
H	9.7889840035	19.6050925243	13.8247365617
H	13.9203475159	15.7604696826	22.9212932781
H	12.4437732899	14.9669086935	23.5279830449
H	12.6110043038	15.3428801969	21.7923824315
H	10.1721624841	17.9329643942	22.9182771401
H	10.4919541536	16.6120983887	21.7565984723
H	10.2253722036	16.2414557139	23.4823803005
H	12.0369395651	18.5665821011	24.6323269443
H	12.0255154808	16.8792593518	25.2021860245
H	13.5466097488	17.6258084355	24.6489135542
H	13.9772781587	11.6849456602	16.5994169066
H	15.0569672759	10.7239720257	15.5497838622
H	15.4299561546	12.4304754132	15.9128105073
H	15.5195865554	12.9334505055	13.3845802274
H	15.1494567491	11.2265793875	13.0287680752
H	14.1364446815	12.5234330680	12.3459987166
H	11.9820366234	11.1338273440	15.1294354233
H	12.0262742502	11.4820921858	13.3854633119
H	13.0162454597	10.1611318236	14.0517333766
H	8.4371369319	14.6800810073	20.0630665425
H	7.5090546851	13.1665626581	19.9239896120
H	8.9744784998	13.2227920254	20.9249634827
H	10.4233198410	11.5144160814	17.9034671542
H	10.4037213268	11.5325158206	19.6771633143
H	8.9198668004	11.0813243788	18.7794985528
H	9.0658063717	13.1569854182	16.5733361163
H	7.5562299583	13.0813433905	17.5148990570
H	8.4514286257	14.6200951293	17.3614004547
H	10.7548368982	22.6663815235	19.0981677217
H	11.7937805516	23.9998854388	19.6652065537
H	11.6009414841	23.6973850793	17.9204402440
H	13.8539383436	20.8825376646	20.3354479309
H	13.1307884303	22.3159683348	21.1111166359
H	12.0911752494	20.9916563103	20.5205583016
H	15.0615437593	22.3430947829	18.6493700068
H	14.1639286266	23.5001302202	17.6424461171
H	14.3661919413	23.8133071867	19.3856722499
H	7.2232686721	20.8090616400	19.0124500573
H	6.6336728542	19.8329626965	17.6599955126
H	5.7425803756	19.8468087371	19.2025455158
H	7.0515004443	16.6304227374	20.0003889813
H	5.6567294904	17.6319443917	19.5683346227
H	6.6501329089	16.8874081586	18.2960396534
H	8.9324476071	19.7850067862	20.4989097865
H	7.5288327192	19.0848100058	21.3468618777
H	8.8798855555	18.0330746178	20.8577912392

Cartesian coordinates of the $Au_{36}(SPh)_{24}$ structural model.

Au	17.7445595511	14.0555725462	20.2591444985
Au	15.5774495478	14.5799374668	18.5681945698
Au	13.9742060959	17.2625503222	18.6986853581
Au	15.9893271214	16.1790103371	20.8310402315
Au	16.3986197262	20.5153573261	21.1369790168
Au	18.0819498496	18.2564560000	21.1375625478
Au	16.3291541189	18.8461631334	18.9521707402
Au	14.4327130705	19.5087956407	17.0106384388
Au	15.8150501623	17.0897109291	16.6117398238
Au	17.8869361126	16.3173561455	18.6625208859
Au	17.1768706440	14.6280219142	16.0837205935
Au	15.1381504259	15.5502788575	14.3824227054
Au	19.8784583690	16.2257587340	16.6657391178
Au	17.6602461833	16.8958295167	14.4918629248
Au	16.7466377154	19.8891085781	14.9064933371
Au	18.4185632562	18.6158823490	16.9089273180
Au	20.2256174917	17.8657046545	18.9396130494
Au	18.7839975352	20.5267643831	19.6814474869
Au	17.2634315841	21.2195038293	17.2910655046
Au	19.3556812796	20.7545933857	15.4456341129
Au	15.6795011913	12.2915534933	21.5466473651
Au	12.7552900130	14.3608417259	20.6312436665
Au	12.9514902723	15.2972424770	16.4328757047
Au	13.9270850384	18.9797393422	21.4023218149
Au	14.5578425438	21.8372510399	19.2497191217
Au	18.3061534769	21.1413962330	23.4727285511
Au	13.6890426332	18.0730112667	14.3024144760
Au	20.2371738248	15.5033448973	20.8921584372
Au	19.5502922599	13.1937313491	18.1409198245
Au	16.5841975347	14.1662381659	12.1615556935
Au	19.8911719053	14.3532909827	13.7104933209
Au	21.2653343818	19.6401577055	21.8628727949
Au	14.7053888737	22.5768326338	15.1909861128
Au	20.8667679045	18.3610723986	14.6418960666
Au	21.4607258099	20.5931708353	17.4312694990
Au	18.2824911970	23.3858977867	14.5098352516
S	13.3246795653	12.2791229785	21.5805039380
S	12.0529123607	16.5033401063	20.0036228478
S	18.0266337716	11.9745160380	21.5726414113
S	13.6547295059	13.4135022569	17.6505028275
S	11.8176298393	17.0201289097	15.2728742852
S	15.0546782958	17.3715147244	22.7146100239
S	12.7627843915	20.5056665885	20.0215749804
S	17.7377432139	12.4028442000	16.8661413358
S	14.2507171439	14.1754788734	12.5151980210
S	13.0524056840	21.0004804482	15.6592079540
S	16.1175140746	21.9478266376	23.1269705468
S	20.3833370664	20.2015433088	23.9606170425
S	19.4548409796	16.8985993367	22.6266834247
S	21.3681971030	14.1335304976	19.3253718361
S	21.2458263392	14.5023185320	15.6100404948
S	18.8395286037	14.3995268541	11.6172895995
S	22.2785544731	18.7510793957	19.9570486227
S	22.5403773949	18.8431027359	16.2458411147
S	19.4175063478	17.5842975191	12.9542076576
S	15.3655217260	19.2033474081	13.0830555193
S	16.1141502314	23.2030196172	18.1354516858

S	20.2643394906	22.1753825257	18.7183042960
S	20.4738623176	22.5794944719	14.1831711195
S	16.1858384407	24.3614276693	14.8286168702
C	12.9618876443	10.9621917156	20.4184835735
C	11.9113938129	11.0626742674	19.4941051556
C	11.5703366356	9.9655203883	18.6964911622
C	12.2684090368	8.7567292052	18.8078621769
C	13.3274628024	8.6679209513	19.7176862173
C	13.6707651322	9.7536101157	20.5238654871
C	10.6861589517	16.3717279060	18.8641180871
C	10.1685364148	15.1477257151	18.4125857037
C	9.1001405156	15.1302361171	17.5102561994
C	8.5652451850	16.3285132284	17.0342659494
C	9.0521404145	17.5480261539	17.5123686516
C	10.1103146959	17.5756105303	18.4232510454
C	18.3697877892	10.6463167852	20.4237888419
C	17.4021969316	10.0829464396	19.5772877427
C	17.7438430073	9.0367502584	18.7138622476
C	19.0587738162	8.5699812079	18.6701820354
C	20.0139698285	9.0856704077	19.5481936939
C	19.6809157196	10.1381188951	20.4106109513
C	14.0027874286	12.0911893094	16.5032885993
C	13.5479960807	12.1189745596	15.1765401292
C	13.6658491136	10.9842182447	14.3690392922
C	14.2245147858	9.8080650543	14.8812188788
C	14.6845325635	9.7841298066	16.2023922223
C	14.5853669935	10.9186182186	17.0111184115
C	11.0311646110	16.1817425190	13.8976619354
C	10.7172839609	16.9110511280	12.7394499883
C	10.0205688290	16.2993682123	11.6963886838
C	9.6385021582	14.9574390523	11.7922738608
C	9.9565542585	14.2295513289	12.9424539130
C	10.6461586138	14.8366940995	13.9950511317
C	13.8185282552	16.2741061549	23.3908687931
C	12.4993865576	16.6949552238	23.6109982039
C	11.5735025289	15.8102334480	24.1693307850
C	11.9589835757	14.5161727773	24.5308214655
C	13.2851964338	14.1119100488	24.3437850958
C	14.2140739025	14.9807193694	23.7693740834
C	11.7281700016	21.4922254399	21.0914399584
C	10.6368852706	20.8511749432	21.7000076428
C	9.7289502909	21.5932795373	22.4571943191
C	9.8975545593	22.9742628526	22.6152051630
C	10.9923563896	23.6073534358	22.0172520706
C	11.9058670222	22.8726166505	21.2554123479
C	18.3429740277	11.6405367476	15.3680217339
C	19.6758880207	11.2443131702	15.1978861658
C	20.0825314112	10.6936176504	13.9787095352
C	19.1662484314	10.5216697756	12.9376686336
C	17.8302934382	10.8911413461	13.1244218808
C	17.4137145079	11.4479015348	14.3332832558
C	13.7155403214	15.2284690267	11.1641864859
C	14.1197442926	16.5641632870	11.0493972895
C	13.6464952520	17.3520062123	10.0011209452
C	12.8012155990	16.8030390596	9.0286045339
C	12.4273975166	15.4583538666	9.1179160405
C	12.8654372737	14.6743528210	10.1942775438

C	11.8434785474	21.7410976172	16.7365024165
C	10.7461613583	20.9329275566	17.0737818137
C	9.7327799014	21.4414071724	17.8863856721
C	9.7961206500	22.7559725997	18.3608418696
C	10.8904728986	23.5579616289	18.0237474597
C	11.9179869634	23.0563825316	17.2189460028
C	15.0283343290	20.9786008075	24.1584379590
C	13.6517858234	21.2401075268	24.0578237601
C	12.7464136151	20.5301208269	24.8511888571
C	13.2018381434	19.5587809602	25.7488842307
C	14.5712959234	19.2871856135	25.8307028310
C	15.4826294801	19.9896871506	25.0407249321
C	21.4249342989	21.4155682628	24.7613030528
C	22.7879716112	21.4982102551	24.4451080481
C	23.6243259173	22.3362437537	25.1868731623
C	23.1152316732	23.0932172814	26.2482857270
C	21.7524236812	23.0139794234	26.5564254584
C	20.9075112537	22.1790359144	25.8203745009
C	18.4566629194	15.9394837103	23.7487421835
C	17.6986436689	16.6303884263	24.7085789315
C	17.0197883110	15.9195018497	25.7014282438
C	17.0680355193	14.5211254815	25.7321345577
C	17.8081728363	13.8343709290	24.7640413188
C	18.5119507503	14.5394598707	23.7849612557
C	22.1053495599	12.8041583411	20.2648222304
C	22.0440761247	12.7364423689	21.6630607069
C	22.7280193895	11.7261465732	22.3443315432
C	23.4775035660	10.7803645775	21.6377702798
C	23.5336780394	10.8463181584	20.2402931080
C	22.8508038038	11.8509407662	19.5525510708
C	22.7613656896	15.2778224898	15.0722231785
C	23.8000996208	15.3415680091	16.0147664594
C	25.0226721671	15.9177072766	15.6633446344
C	25.2179616302	16.4309822353	14.3766417912
C	24.1843020674	16.3626064925	13.4393212207
C	22.9569289905	15.7902673101	13.7805420024
C	19.3946478743	12.9739401743	10.6982645119
C	18.5263744135	12.3077356014	9.8233491124
C	19.0177619364	11.3094091335	8.9768291657
C	20.3709396207	10.9556298821	9.0108225339
C	21.2280458155	11.5977184336	9.9138398905
C	20.7488857428	12.6083720095	10.7490877509
C	23.2085954670	17.3132733914	20.4695934044
C	23.2605036875	16.8465443759	21.7920163708
C	23.9850357794	15.6901099739	22.0916768846
C	24.6558399923	14.9925304765	21.0832206980
C	24.6170887755	15.4716560862	19.7699334229
C	23.9011051027	16.6286929266	19.4582511774
C	23.9525721152	19.5195343807	15.3825334047
C	25.1332235313	19.6797562140	16.1269280984
C	26.3122121636	20.0841080574	15.4920753250
C	26.3181746004	20.3444076552	14.1156332449
C	25.1342571708	20.2043080534	13.3812977657
C	23.9556824744	19.7880049784	14.0069985534
C	18.8679255206	18.9211173425	11.9133136035
C	19.5051688134	20.1683296486	11.8988383395
C	19.1064007216	21.1491197560	10.9870976028
C	18.0846624474	20.8824819394	10.0706284226
C	17.4565632707	19.6316519413	10.0780367928

C	17.8374205135	18.6512948891	10.9965654304
C	14.5353696001	20.5738437516	12.2966921467
C	15.2812450507	21.6952414806	11.9022857789
C	14.6634539785	22.7092767461	11.1682172379
C	13.3065217116	22.6282847560	10.8419950692
C	12.5628818083	21.5155644332	11.2445843827
C	13.1747443870	20.4876192129	11.9639135121
C	17.0537094320	24.2279447388	19.2543036584
C	17.9684029459	25.1313011929	18.6822164569
C	18.5544299844	26.1307222307	19.4690375456
C	18.2628507470	26.2138521826	20.8367002970
C	17.3953245613	25.2754787291	21.4130522366
C	16.7828821970	24.2957988249	20.6271321196
C	21.3036304766	22.7398571434	20.0558741898
C	22.7009512379	22.7416361786	19.9493862890
C	23.4721504590	23.3084543098	20.9678600619
C	22.8578338246	23.8657087488	22.0925123281
C	21.4627817375	23.8477724889	22.2022778615
C	20.6822752948	23.2926109472	21.1884023169
C	21.5346486124	23.4230808456	15.3431197668
C	22.8507176992	22.9453856384	15.4617017192
C	23.7449392923	23.5696332818	16.3344771705
C	23.3393190852	24.6732106696	17.0910010859
C	22.0302859396	25.1522534641	16.9695306530
C	21.1277620022	24.5284579777	16.1046884057
C	15.7045034552	25.1160971086	13.2800304002
C	14.3532653585	25.4436652150	13.0881958633
C	13.9513855825	26.0900992893	11.9166939309
C	14.8865942053	26.3777951356	10.9180567216
C	16.2398176453	26.0975509444	11.1295003946
C	16.6496711349	25.4528751652	12.3012674906
H	11.3724025143	12.0024502048	19.3975036563
H	10.7571046873	10.0644173407	17.9806844937
H	12.0026291501	7.8921246434	18.1989858175
H	13.8894959118	7.7396951772	19.8102148920
H	14.4881652215	9.6648911917	21.2375489804
H	10.6191226483	14.2203855127	18.7626656243
H	8.7238214721	14.1743323417	17.1539108453
H	7.7308000854	16.3118206046	16.3320069325
H	8.6472838322	18.4883080488	17.1458027786
H	10.5177030440	18.5240088295	18.7706315055
H	16.3868334507	10.4744033133	19.5993510890
H	16.9892087114	8.6430698252	18.0372727548
H	19.3278339508	7.7677520123	17.9841518431
H	21.0341798882	8.7088432813	19.5417615943
H	20.4332102759	10.5645817872	21.0705881944
H	13.0960188122	13.0266132413	14.7821269245
H	13.3143159251	11.0316159238	13.3402665123
H	14.2992938434	8.9077422704	14.2698398470
H	15.1037758809	8.8625191078	16.6077564899
H	14.9220374955	10.8931270346	18.0440902104
H	11.0271126693	17.9507455832	12.6575074130
H	9.7931030332	16.8708906673	10.7998832577
H	9.0815824413	14.4886052441	10.9829456840
H	9.6736673284	13.1826943395	13.0262438197
H	10.8875050525	14.2762311938	14.8973367512
H	12.2060989041	17.7039976427	23.3256909868

H	10.5449903592	16.1348938089	24.3128360029
H	11.2310971549	13.8246744066	24.9491264236
H	13.5982630921	13.1070441744	24.6192143265
H	15.2379461216	14.6540079681	23.5939821203
H	10.5044366664	19.7778434031	21.5724541449
H	8.8780222852	21.0928838448	22.9158843178
H	9.1757118886	23.5467005303	23.1951533110
H	11.1372894368	24.6789452547	22.1373456199
H	12.7555597552	23.3599072419	20.7776483444
H	20.3890400670	11.4030462210	16.0052508776
H	21.1258610774	10.4188021838	13.8392030492
H	19.4933650987	10.1281573356	11.9780680810
H	17.1111165443	10.7768498652	12.3158911807
H	16.3811947348	11.7641679240	14.4669659114
H	14.8052342070	16.9823137874	11.7805457591
H	13.9445892535	18.3976906960	9.9471168662
H	12.4321659427	17.4255467612	8.2191626646
H	11.7832497189	15.0176817801	8.3593154855
H	12.5572443829	13.6350866726	10.2821186645
H	10.7044979204	19.9108858826	16.7010988363
H	8.8850406269	20.8099471455	18.1491927459
H	8.9985000032	23.1434538920	18.9927270812
H	10.9537916748	24.5780136884	18.3966259437
H	12.7783016039	23.6730961828	16.9616878911
H	13.2905184199	21.9910499361	23.3597378862
H	11.6842937062	20.7451603294	24.7554421819
H	12.5035789119	19.0185019088	26.3864787056
H	14.9340159790	18.5202085334	26.5123199355
H	16.5495380759	19.7792415278	25.0998120345
H	23.1802383380	20.9195314652	23.6097155277
H	24.6793487400	22.3966029182	24.9272633231
H	23.7729396800	23.7540928598	26.8059739362
H	21.3490098625	23.5831004363	27.3939629685
H	19.8498607496	22.1099731326	26.0686236760
H	17.6511118952	17.7168823463	24.6710416616
H	16.4447444846	16.4594871477	26.4519189812
H	16.5242730319	13.9741916525	26.4993026536
H	17.8476946496	12.7469222720	24.7638226828
H	19.1072224059	14.0062754024	23.0474639348
H	21.4667769327	13.4836975925	22.2070224287
H	22.6744124995	11.6821039188	23.4302946614
H	24.0120607550	9.9967794366	22.1697544607
H	24.1143778033	10.1152898671	19.6820464933
H	22.8889238090	11.9034475453	18.4659854026
H	23.6329577085	14.9489013368	17.0161819126
H	25.8297723445	15.9692438332	16.3931137950
H	26.1690824129	16.8897995110	14.1130404356
H	24.3251782004	16.7674117492	12.4395584982
H	22.1446907733	15.7395587990	13.0562760646
H	17.4727591155	12.5834233257	9.8088166443
H	18.3363536266	10.8052994954	8.2951239569
H	20.7518240987	10.1832020404	8.3464746292
H	22.2783693641	11.3187136854	9.9640263931
H	21.4142040420	13.1107702904	11.4496818978
H	22.7176501578	17.3810364637	22.5706249905
H	24.0064724142	15.3239333635	23.1160353056
H	25.1858808407	14.0716389259	21.3144016753
H	25.1338654316	14.9312666498	18.9790825536
H	23.8417949697	16.9862042633	18.4313516108

H	25.1265377832	19.4642546203	17.1940469251
H	27.2353722703	20.1828210981	16.0642691327
H	27.2440809816	20.6463766431	13.6259555023
H	25.1274590015	20.4092178213	12.3127503954
H	23.0370295217	19.6552106662	13.4357305673
H	20.3152410917	20.3634457124	12.5972465629
H	19.6045225666	22.1166024977	10.9979027851
H	17.7806255756	21.6352884309	9.3454515708
H	16.6667609389	19.4166447405	9.3600160384
H	17.3453765330	17.6799680147	11.0049758664
H	16.3331261336	21.7717089439	12.1725469425
H	15.2418923200	23.5785322923	10.8670873382
H	12.8326266858	23.4351629839	10.2878944813
H	11.5041824236	21.4421220622	11.0047021097
H	12.6050357429	19.6156410922	12.2812933673
H	18.1846488906	25.0723674672	17.6166176879
H	19.2507178694	26.8276071066	19.0095254321
H	18.7017428016	27.0085597586	21.4391120884
H	17.1661955144	25.3125838540	22.4764309376
H	16.0703909459	23.6026616686	21.0670566290
H	23.1703635336	22.3054623232	19.0694169460
H	24.5565840067	23.3078779490	20.8794322198
H	23.4588070483	24.2924723905	22.8915521422
H	20.9793518855	24.2515191678	23.0893778650
H	19.5964159197	23.2778895948	21.2732921632
H	23.1716992379	22.0885816293	14.8742713774
H	24.7574417672	23.1800661591	16.4198138877
H	24.0342689296	25.1571589680	17.7732790300
H	21.7112209694	26.0237256998	17.5427688334
H	20.1061973804	24.8934806488	16.0103671302
H	13.6221165048	25.1736288212	13.8480265755
H	12.8960273198	26.3066495506	11.7644973299
H	14.5682533076	26.8553225267	9.9941464432
H	16.9767833681	26.3231772361	10.3616767959
H	17.6961446983	25.1935893729	12.4587909602

References

Dass, A., Jones, T., Rambukwella, M., Crasto, D., Gagnon, K.J., Sementa, L., et al. (2016). Crystal Structure and Theoretical Analysis of Green Gold Au30(S-tBu)18 Nanomolecules and Their Relation to Au30S(S-tBu)18. *The Journal of Physical Chemistry C* 120(11)**,** 6256-6261. doi: 10.1021/acs.jpcc.6b00062.

Goedecker, S., Teter, M., and Hutter, J. (1996). Separable dual-space Gaussian pseudopotentials. *Physical Review B* 54(3)**,** 1703-1710.

Grimme, S., Antony, J., Ehrlich, S., and Krieg, H. (2010). A consistent and accurate ab initio parametrization of density functional dispersion correction (DFT-D) for the 94 elements H-Pu. *The Journal of Chemical Physics* 132(15)**,** 154104. doi: 10.1063/1.3382344.

Hutter, J., Iannuzzi, M., Schiffmann, F., and VandeVondele, J. (2014). cp2k: atomistic simulations of condensed matter systems. *Wiley Interdisciplinary Reviews: Computational Molecular Science* 4(1)**,** 15-25. doi: 10.1002/wcms.1159.

Lippert, G., Hutter, J., and Parrinello, M. (1999). The Gaussian and augmented-plane-wave density functional method for ab initio molecular dynamics simulations. *Theoretical Chemistry Accounts* 103(2)**,** 124-140. doi: 10.1007/s002140050523.

Martyna, G.J., Klein, M.L., and Tuckerman, M. (1992). Nosé–Hoover chains: The canonical ensemble via continuous dynamics. *The Journal of Chemical Physics* 97(4), 2635-2643. doi: doi:http://dx.doi.org/10.1063/1.463940.

Nimmala, P.R., Knoppe, S., Jupally, V.R., Delcamp, J.H., Aikens, C.M., and Dass, A. (2014). Au36(SPh)24 Nanomolecules: X-ray Crystal Structure, Optical Spectroscopy, Electrochemistry, and Theoretical Analysis. *The Journal of Physical Chemistry B* 118(49), 14157-14167. doi: 10.1021/jp506508x.

Perdew, J.P., Burke, K., and Ernzerhof, M. (1996). Generalized Gradient Approximation Made Simple. *Physical Review Letters* 77(18), 3865-3868.

Qian, H., Eckenhoff, W.T., Zhu, Y., Pintauer, T., and Jin, R. (2010). Total Structure Determination of Thiolate-Protected Au38 Nanoparticles. *Journal of the American Chemical Society* 132(24), 8280-8281. doi: 10.1021/ja103592z.

VandeVondele, J., and Hutter, J. (2007). Gaussian basis sets for accurate calculations on molecular systems in gas and condensed phases. *The Journal of Chemical Physics* 127(11), 114105. doi: 10.1063/1.2770708.
